# Supplementary material for: Conjugated dual size effect of core-shell particles synergizes bimetallic catalysis
Source: Nat Commun. 2023 Feb 1;14:530. doi: 10.1038/s41467-023-36147-2 (PMC9892500; doi:10.1038/s41467-023-36147-2)
Supplement: Supplementary file 1 — Supplementary Information [file 41467_2023_36147_MOESM1_ESM.pdf]

Supporting Information

Conjugated dual size effect of core-shell particles synergies bimetallic catalysis

Xiaohui Zhang,[1,¶] Zhihu Sun,[2,¶] Rui Jin,[1,¶] Chuwei Zhu,[1,¶] Chuanlin Zhao,[1] Yue Lin,[1] Qiaoqiao Guan,[1] Lina Cao,[1] Hengwei Wang,[1] Shang Li,[1] Hancheng Yu,[1] Xinyu Liu,[1] Leilei Wang,[1] ShiqiangWei,[2,*] Wei-Xue Li,[1,*] Junling Lu,[1,*]

[1]Department of Chemical Physics, Hefei National Research Center for Physical Sciences at the Microscale, Key Laboratory of Surface and Interface Chemistry and Energy Catalysis of Anhui Higher Education Institutes, Collaborative Innovation Center of Chemistry for Energy Materials (*i*ChEM), University of Science and Technology of China, Hefei, 230026, China

[2]National Synchrotron Radiation Laboratory, University of Science and Technology of China, Hefei, 230029, China

[¶]These authors contributed equally: Xiaohui Zhang, Zhihu Sun, Rui Jin and Chuwei Zhu.

*To whom correspondence should be addressed: junling@ustc.edu.cn, sqwei@ustc.edu.cn and wxli70@ustc.edu.cn

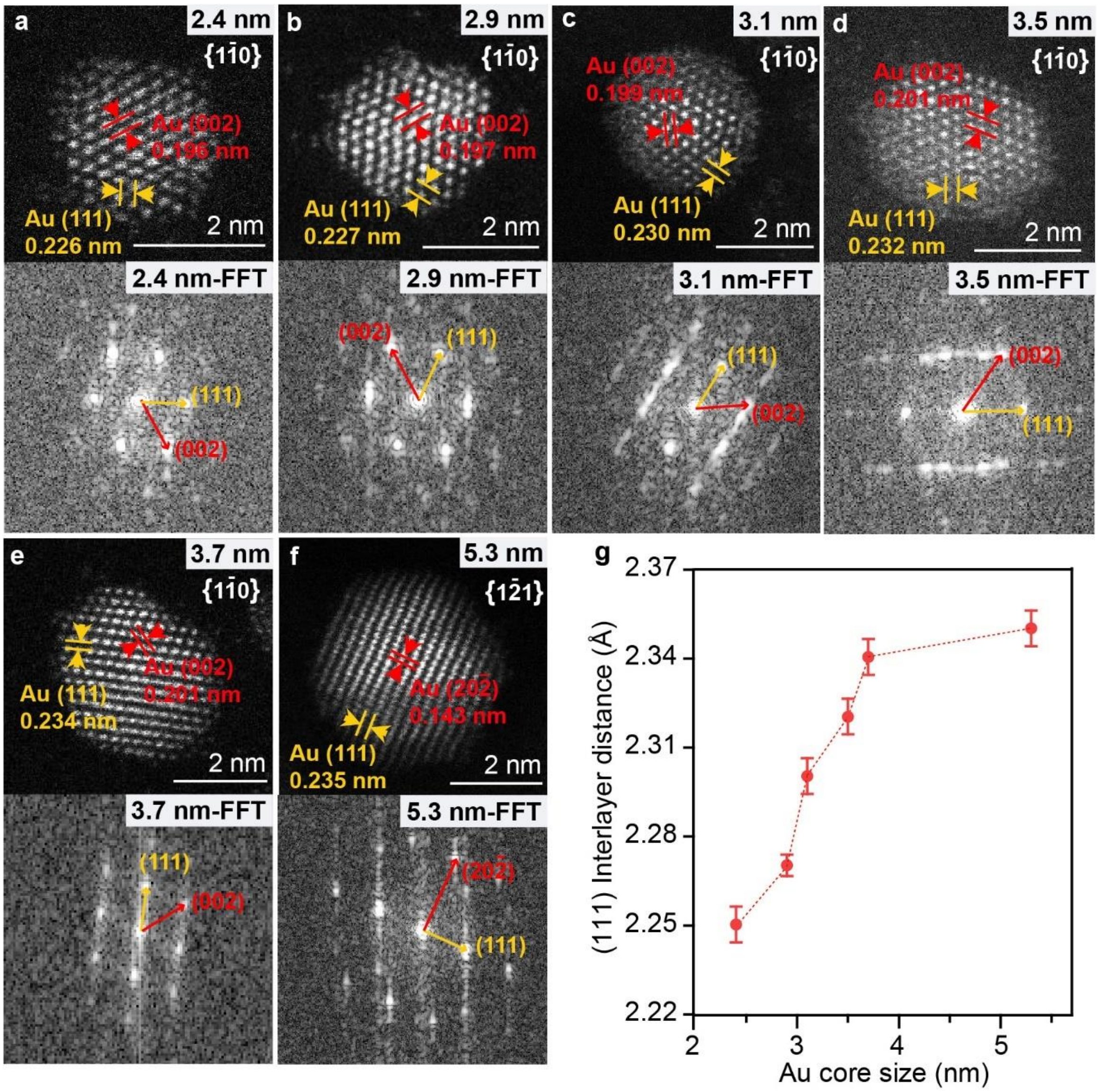

Supplementary Figure 1 | Analysis of Au-Au plane distances in different size of Au particles. a-f, Representative atomic-resolution HAADF-STEM images and their FFT results of Au NPs/SiO_2 in different sizes as indicated in the STEM images. The lattice distances of different facets were displayed by yellow and red. The zone axes are $\{1\bar{1}0\}$ (**a-e**) and $\{1\bar{2}1\}$ (**f**) labelled in the images. **g**, The Au (111) interlayer distance as a plot to Au cluster size. Error bars represent the standard deviation from the measurements at different locations.

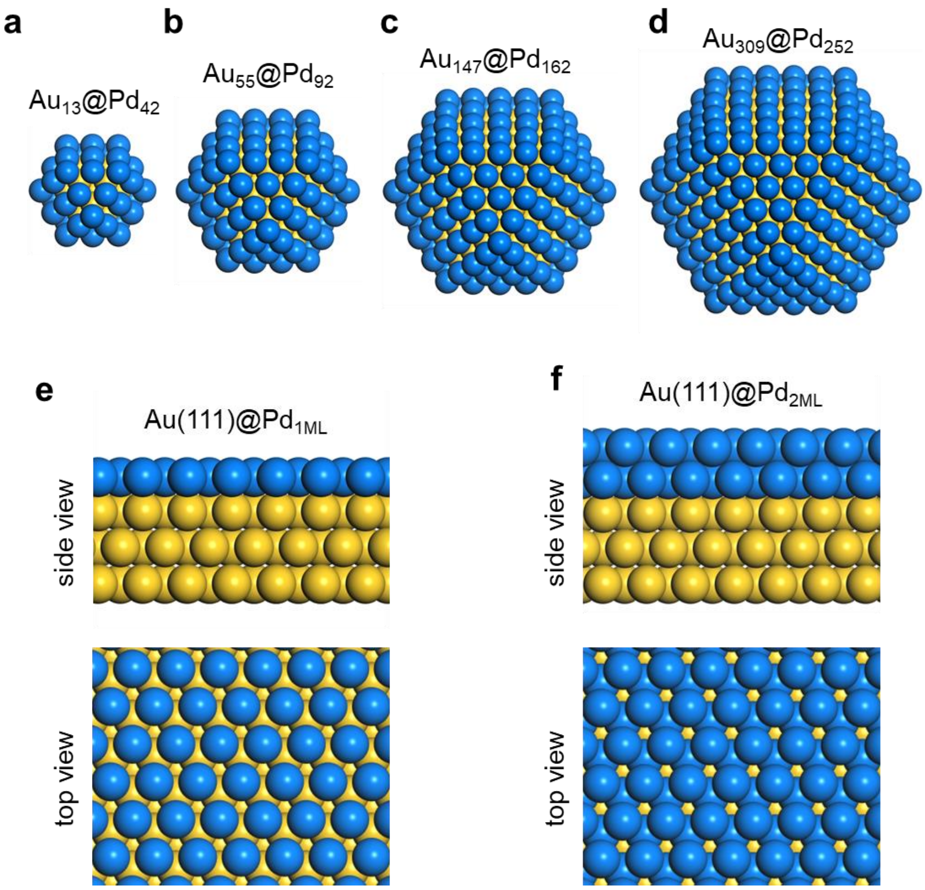

Supplementary Figure 2 | Computational models of Au@Pd core-shell models. Cubooctahedral Au@Pd_{1ML} core-shell clusters of Au_{13}@Pd_{42} (**a**), Au_{55}@Pd_{92} (**b**), Au_{147}@Pd_{162} (**c**) and Au_{309}@Pd_{252} (**d**) constructed by substituting the outermost atom layer with Pd. (**e**, **f**) Au(111)@Pd_{nML} slab models. Here, the blue and gold spheres represent Pd and Au atoms, respectively.

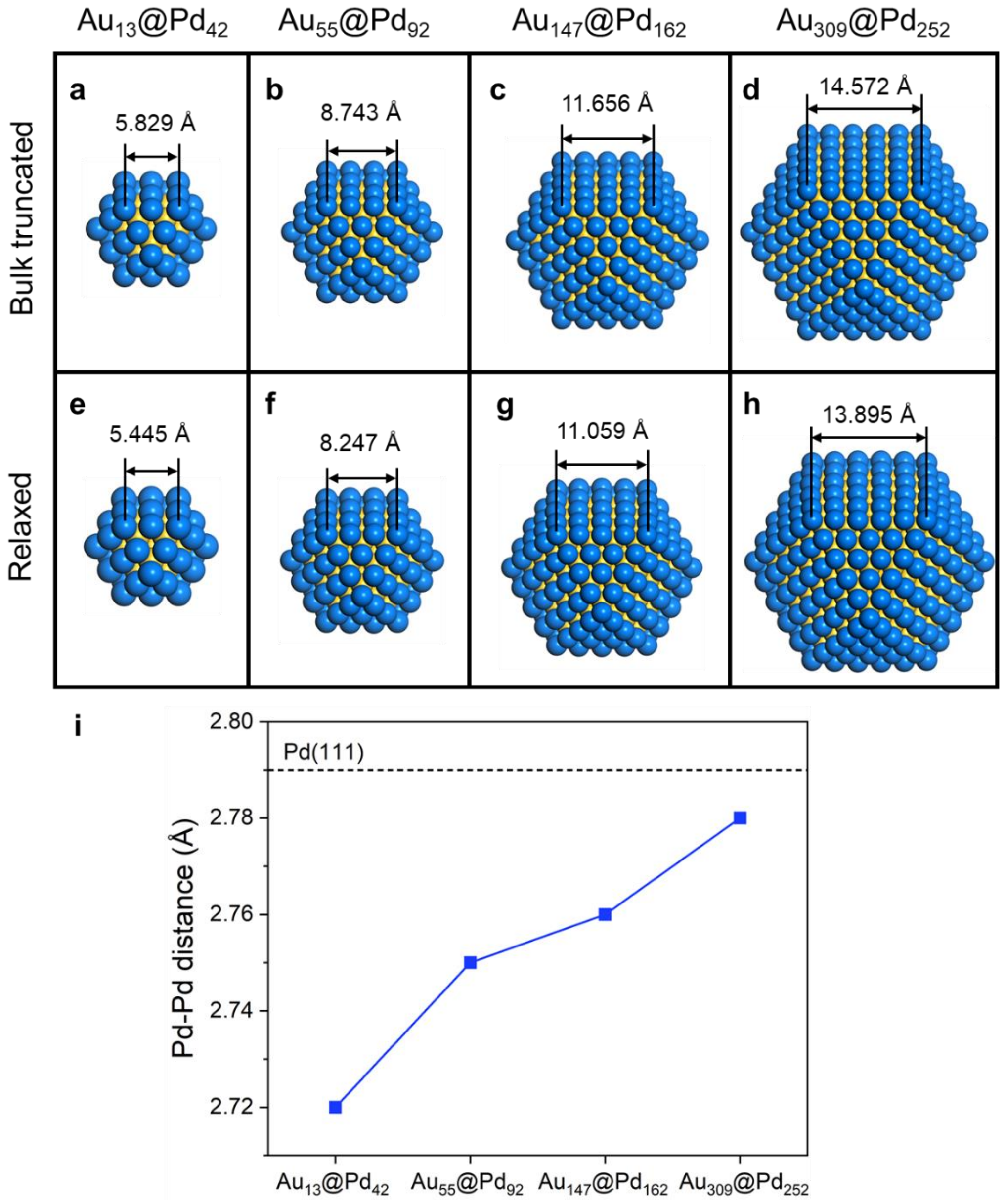

Supplementary Figure 3 | Structure models of cubooctahedral Au@Pd$_{1ML}$ core-shell clusters. Bulk truncated $Au_{13}@Pd_{42}$ (**a**), $Au_{55}@Pd_{92}$ (**b**), $Au_{147}@Pd_{162}$ (**c**), and $Au_{309}@Pd_{252}$ (**d**) clusters. Relaxed $Au_{13}@Pd_{42}$ (**e**), $Au_{55}@Pd_{92}$ (**f**), $Au_{147}@Pd_{162}$ (**g**), and $Au_{309}@Pd_{252}$ (**h**) clusters. The corresponding edge lengths of Pd(111) facet before and after relaxation are indicated. The blue and gold spheres represent Pd and Au atoms, respectively. **i**) Average Pd-Pd distances in the relaxed clusters.

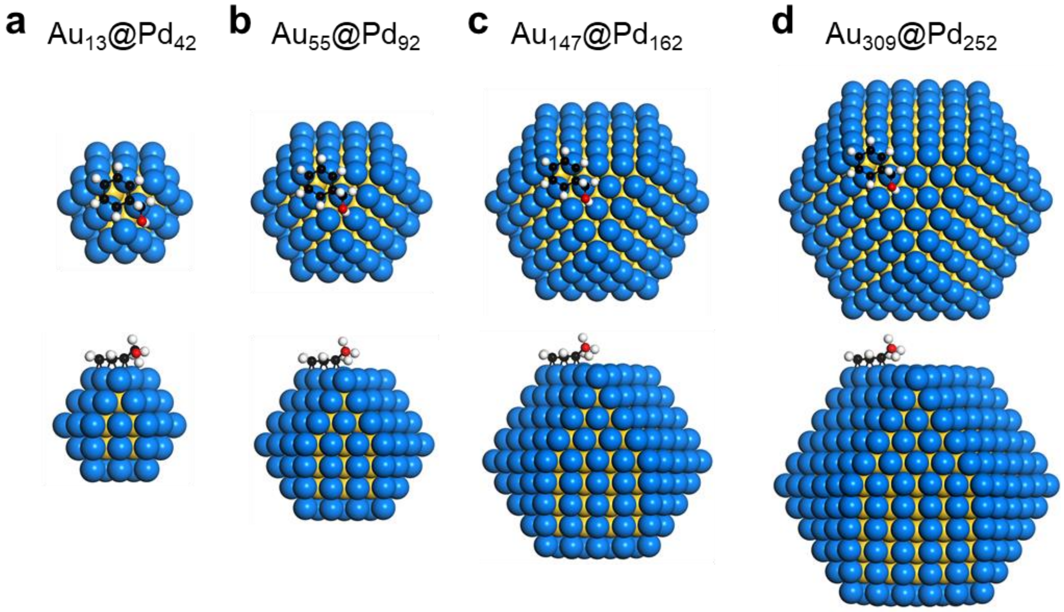

Supplementary Figure 4 | Top and side views of adsorption geometries of BzOH on Au@Pd core-shell clusters. Adsorption of BzOH on $Au_{13}@Pd_{42}$ (**a**), $Au_{55}@Pd_{92}$ (**b**), $Au_{147}@Pd_{162}$ (**c**) and $Au_{309}@Pd_{252}$ (**d**). The blue, gold, black, red, and white spheres represent Pd, Au, C, O, and H atoms, respectively.

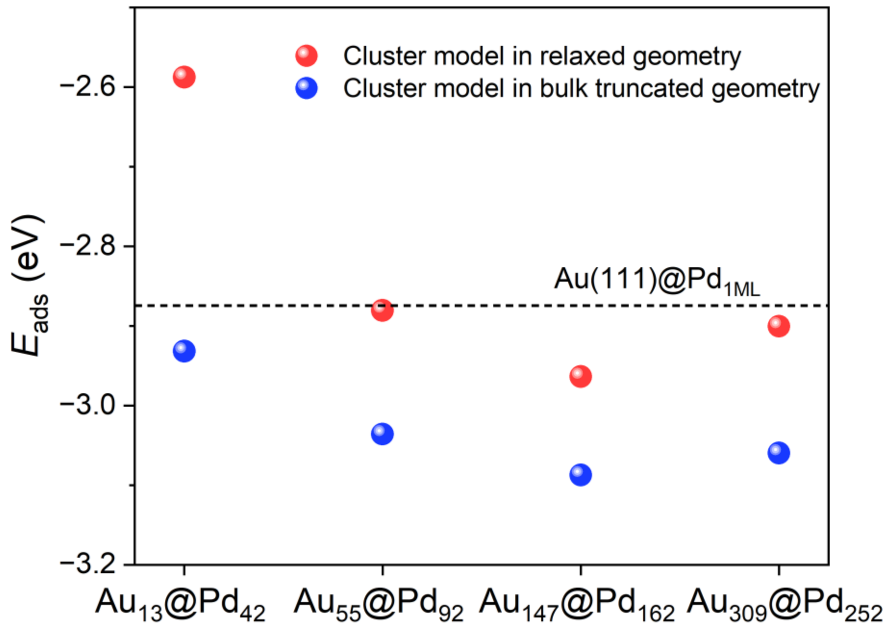

Supplementary Figure 5 | Adsorption energies of BzOH on Au@Pd$_{1ML}$ core-shell cluster models in bulk truncated (blue) and relaxed geometry (red). The adsorption of BzOH on Au(111)@Pd$_{1ML}$ as reference is indicated.

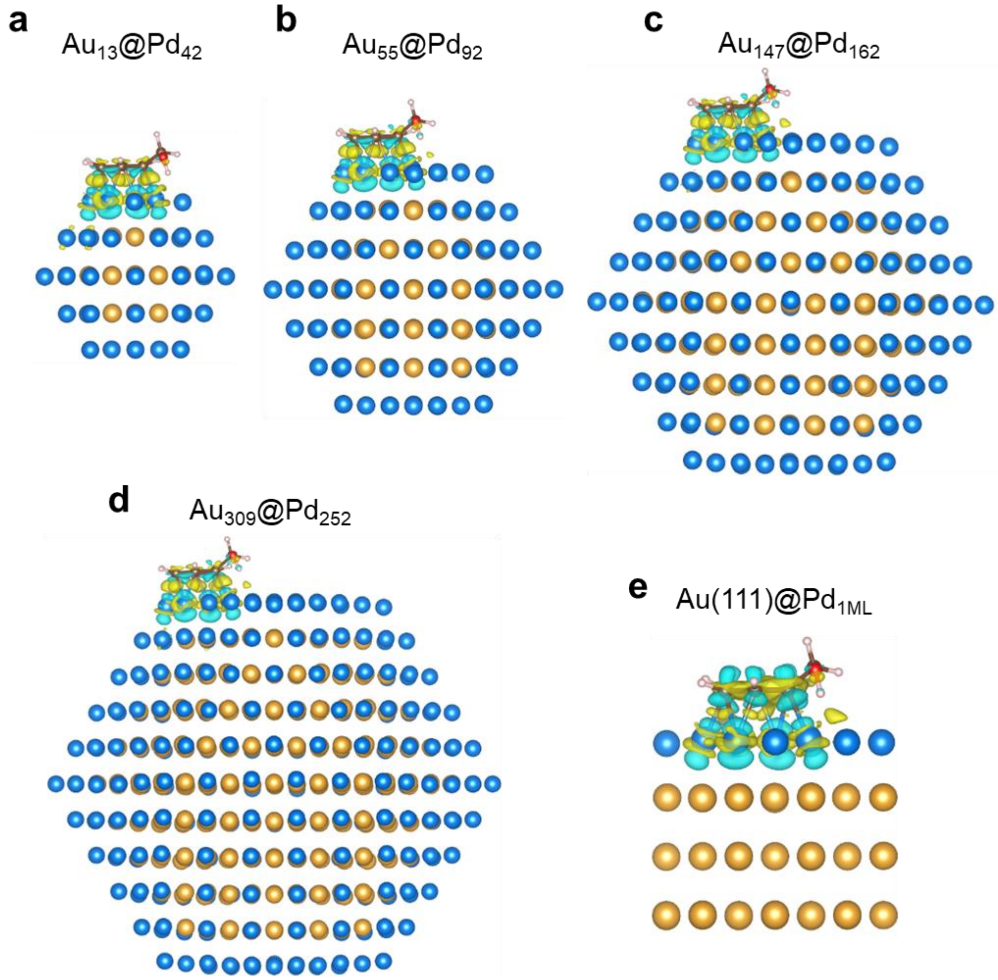

Supplementary Figure 6 | Charge density difference contours upon BzOH adsorption $Au@Pd_{1ML}$ clusters. **a**, $Au_{13}@Pd_{42}$. **b**, $Au_{55}@Pd_{92}$. **c**, $Au_{147}@Pd_{162}$. **d**, $Au_{309}@Pd_{252}$. **e**, $Au(111)@Pd_{1ML}$.The blue and gold spheres are Pd and Au atoms, while the areas of cyan and yellow contours represent the depletion and accumulation of electrons by 0.005 e/Å^3, respectively.

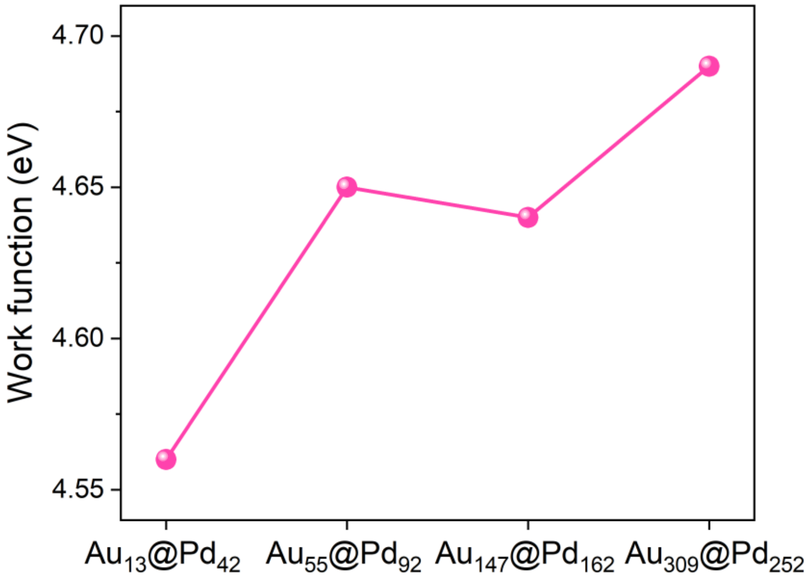

Supplementary Figure 7 | Work functions of Au@Pd$_{1ML}$ core-shell clusters in different sizes.

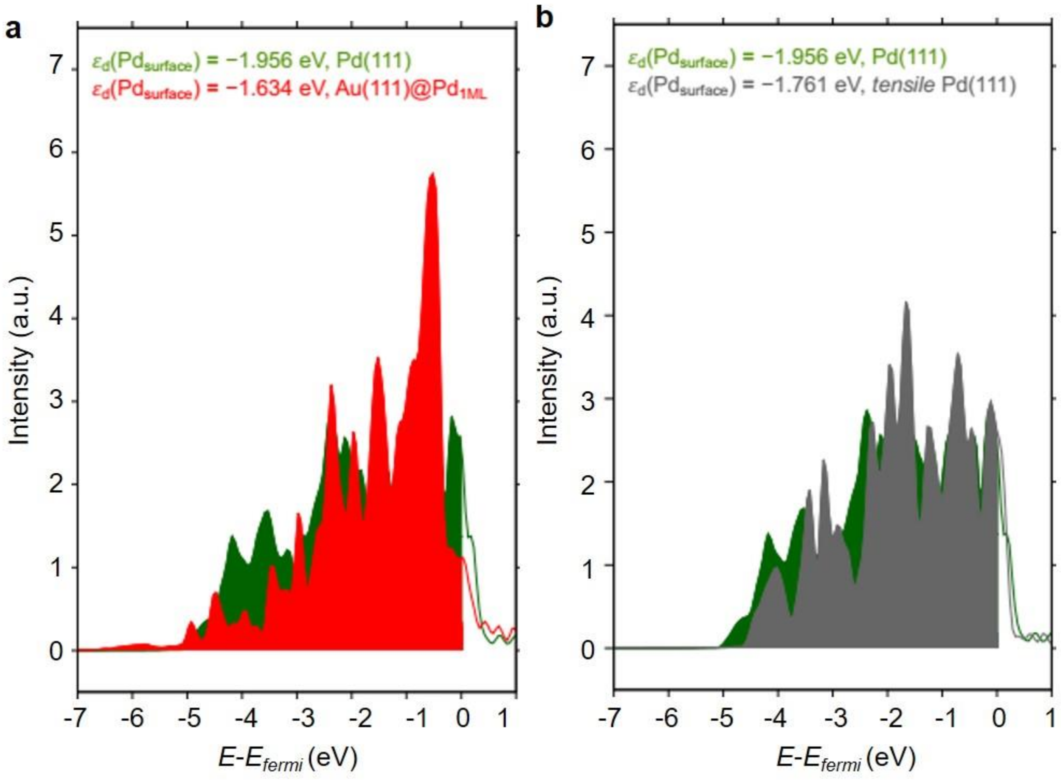

Supplementary Figure 8 | Projected density of states. a, Surface Pd 4*d* band for pristine 4-layer Pd(111) (green) and 3-layer Au(111)@Pd_{1ML} (red); **b**, Surface Pd 4*d* for pristine 4-layer Pd(111) (green) and 4-layer tensile Pd(111) in Au lattice constant (grey). Here $\mathcal{E}_d$ is filled *d*-band center.

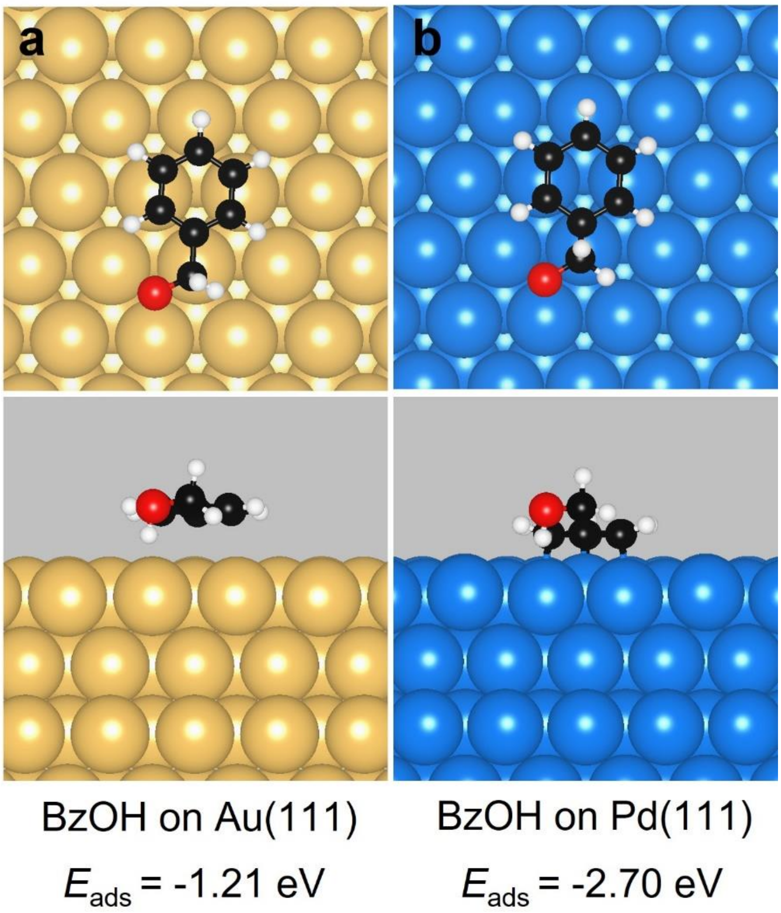

Supplementary Figure 9 | Top and side views of adsorption geometries of BzOH on Au(111) and Pd(111) slab model surfaces. a, BzOH adsorption on Au(111). **b**, BzOH adsorption on 4-fold bridge sites of Pd(111). The blue, gold, black, red, and white spheres represent Pd, Au, C, O, and H atoms, respectively.

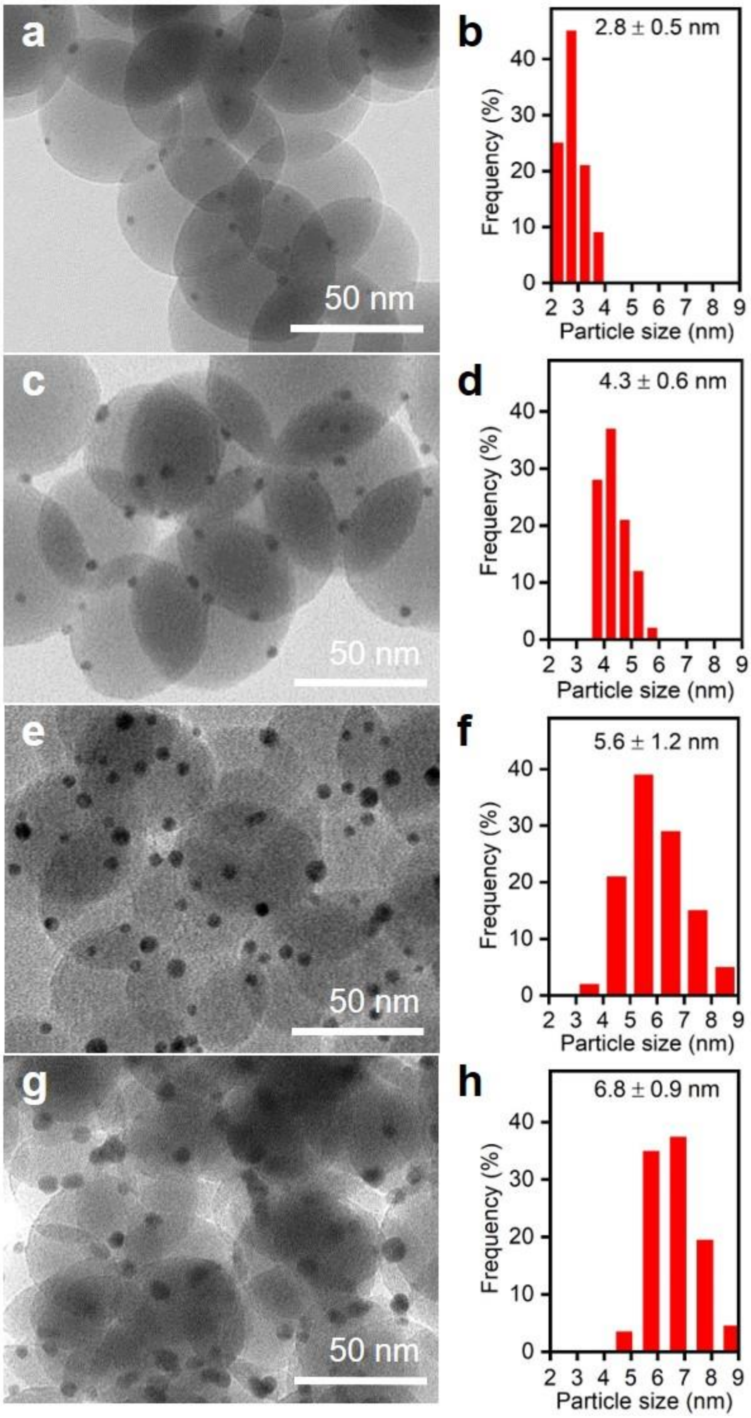

Supplementary Figure 10 | Representative TEM images of *x* nm-Au catalysts and its corresponding histogram of particle size distribution. a,b, 2.8 nm-Au; **c,d**, 4.3 nm-Au; **e,f**, 5.6 nm-Au; **g,h**, 6.8 nm-Au.

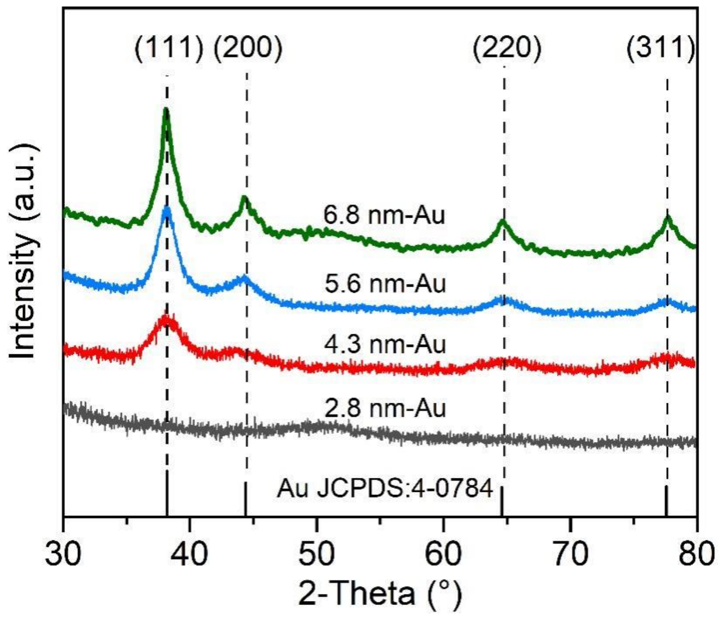

Supplementary Figure 11 | XRD patterns of *x* nm-Au catalysts with different particle size.

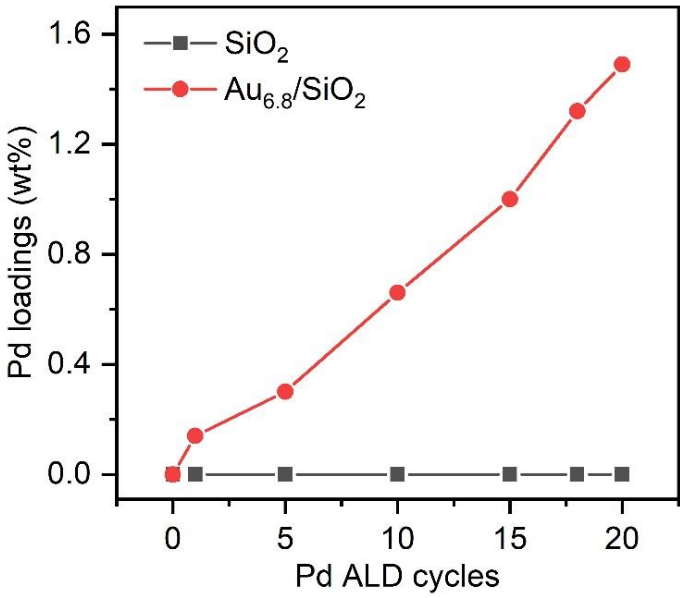

Supplementary Figure 12 | The Pd contents after depositing Pd on $Au_{6.8}/SiO_2$ and bare SiO_2 support for different number of Pd ALD cycles as determined by ICP-AES.

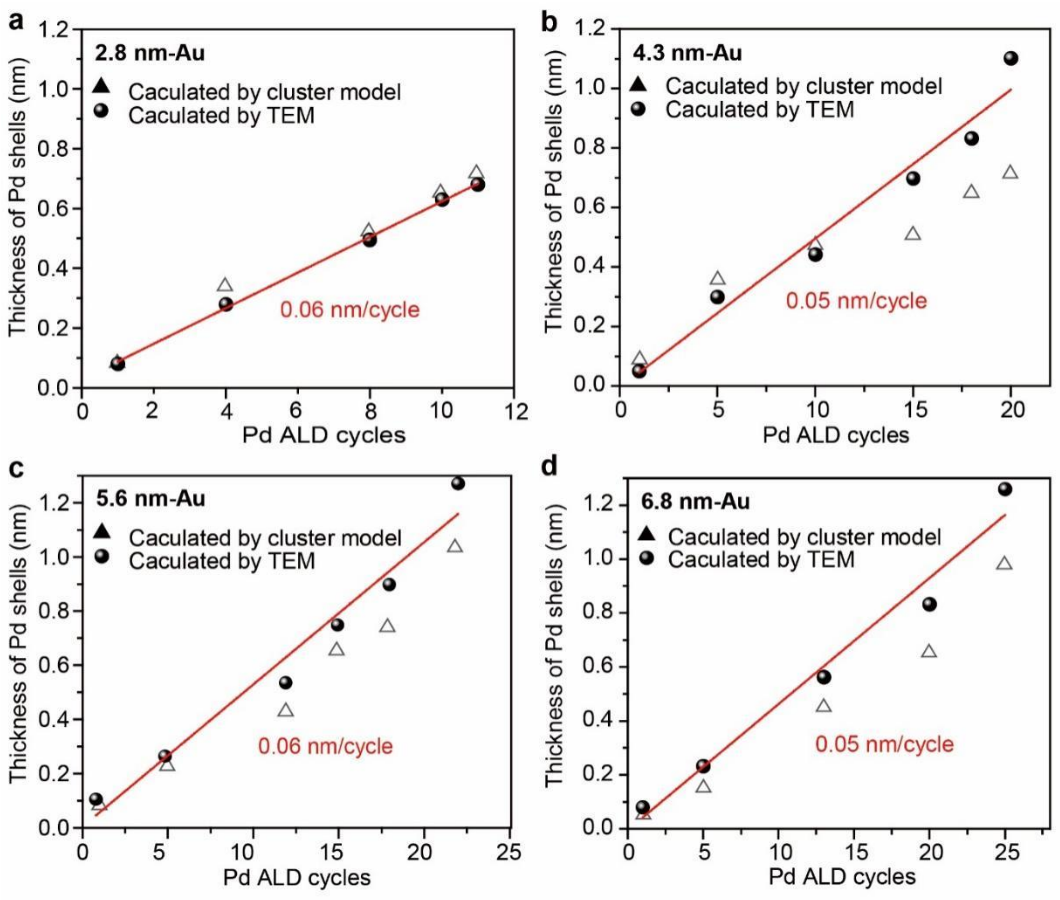

Supplementary Figure 13 | Particle size variation after Pd deposition according to cuboctahedron cluster model and TEM. Particle size variation after depositing Pd on 2.8 nm-Au (**a**), 4.3 nm-Au (**b**), 5.6 nm-Au (**c**), and 6.8 nm-Au (**d**) for different number of Pd ALD cycles. The growth rates of Pd shell on *x* nm-Au as the number of Pd ALD cycles are indicated.

Supplementary Note:

We estimated the thicknesses of Pd shells in these Au@Pd core-shell catalysts in two ways: One way is to measure the particle size of Au@Pd core-shell particles after different numbers of cycles of Pd ALD using TEM. Then the Pd shell thickness is obtained by taking the halves of the differences between the sizes of Au@Pd and the corresponding bare Au particles. The other way is to use the ‘‘cuboctahedron cluster’’ model and the corresponding Pd contents as determined by ICP-AES (Supplementary Table 4-5). We found that these two ways consist very well with each other (Supplementary Fig. 13). Additionally, we found that the Pd deposition rates on Au NPs with different particle sizes are similar, about 0.05~0.06 nm per cycle (or 0.22-0.26 ML per cycle).

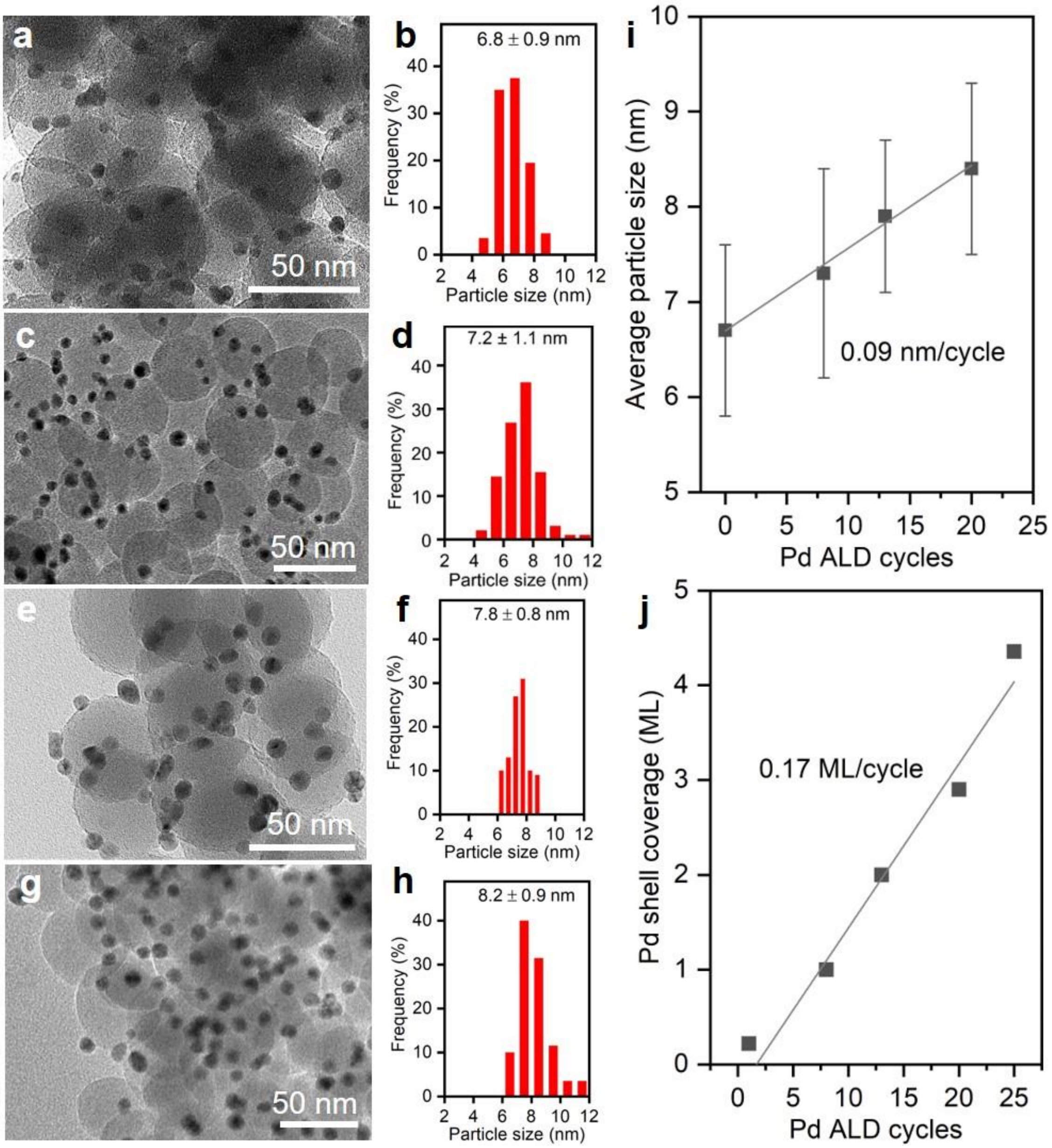

Supplementary Figure 14 | Representative TEM images of 6.8 nm-Au and $Au_{6.8}$@yML-Pd/SiO_2 as well as their corresponding particle size distributions. **a**,**b**, 6.8 nm-Au; **c**,**d**, $Au_{6.8}$@1.1ML-Pd; **e**,**f**, $Au_{6.8}$@2ML-Pd; **g**,**h**, $Au_{6.8}$@2.9ML-Pd. **i**, Particle size variation of $Au_{6.8}$@Pd core-shell particles after different numbers of cycles of Pd ALD. Error bars represent the standard deviation. **j**, Pd shell thicknesses $Au_{6.8}$@Pd core-shell particles after different numbers of cycles of Pd ALD. It should be noted that the growth rate of Pd shell is half of the growth rate of particle size (0.09 nm/cycle), about ~0.05 nm/cycle.

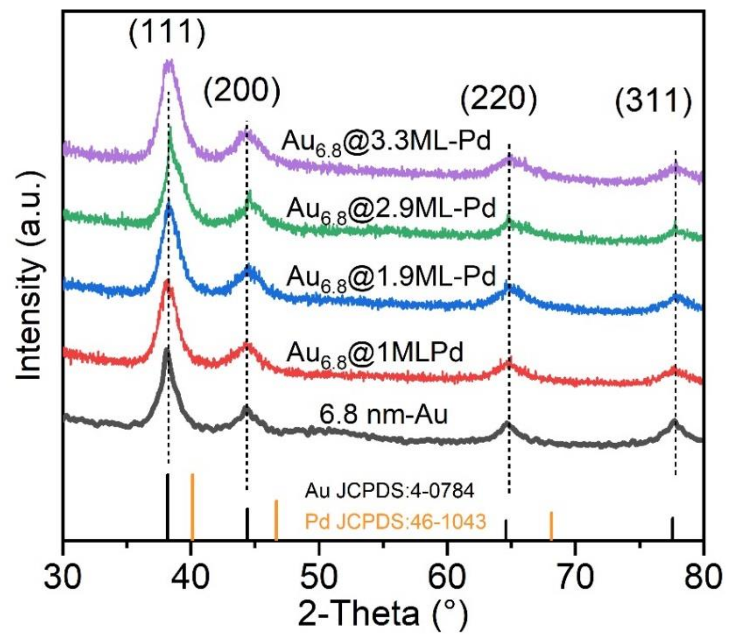

Supplementary Figure 15 | XRD patterns of $Au_{6.8}$@yML-Pd catalysts.

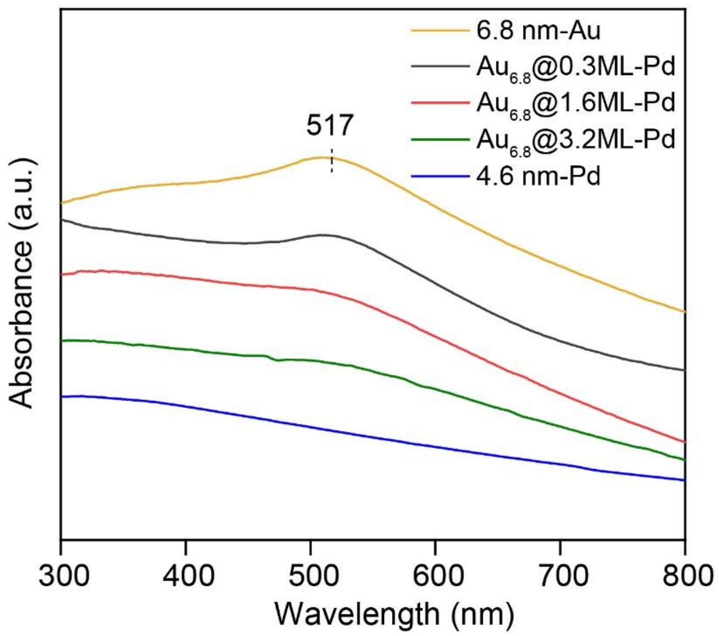

Supplementary Figure 16 | UV–Vis spectra of 6.8 nm-Au, 4.6 nm-Pd, and $Au_{6.8}$@yML-Pd catalysts.

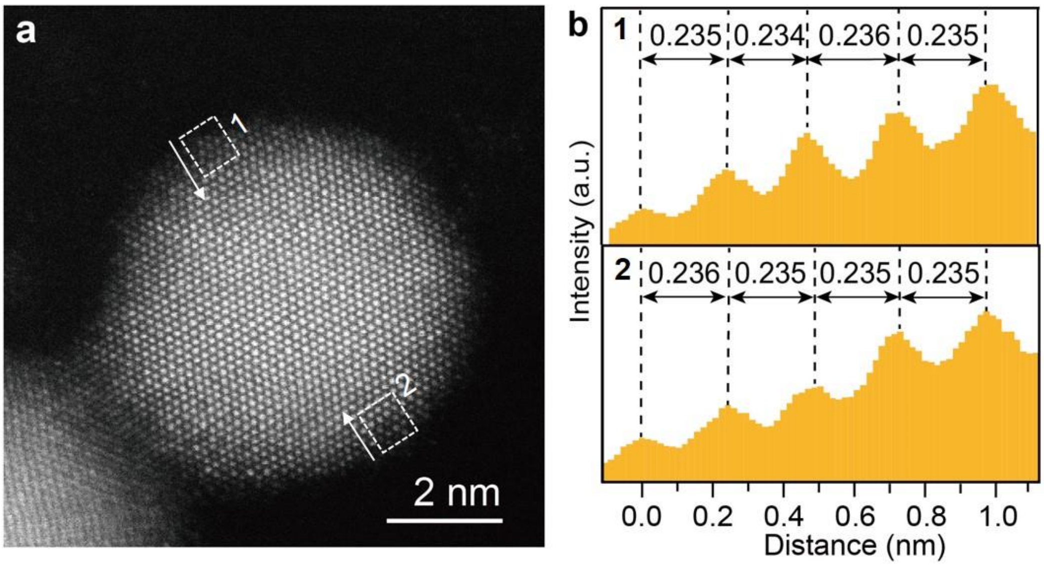

Supplementary Figure 17 | Morphology of the 6.8 nm-Au monometallic catalyst. a, A representative atomic-resolution HAADF-STEM image of one Au nanoparticle in the 6.8 nm-Au sample. **b**, The corresponding line intensity profiles along the numbered dashed rectangles, where the interplanar distances are indicated.

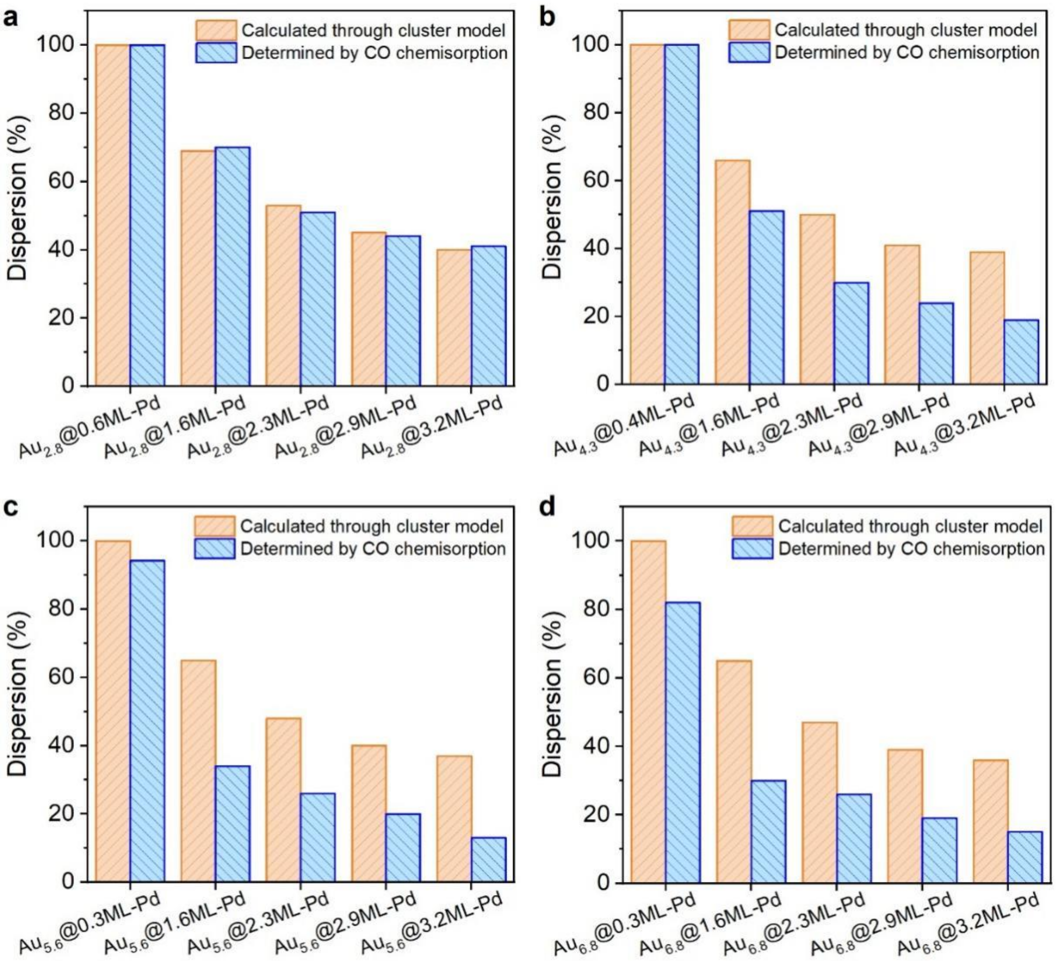

Supplementary Figure 18 | Comparison of the Pd dispersions by the two methods of cluster model and CO chemisorption. a, $Au_{2.8}$@*y*ML-Pd. **b,** $Au_{4.3}$@*y*ML-Pd. **c,** $Au_{5.6}$@*y*ML-Pd. **d,** $Au_{6.8}$@*y*ML-Pd.

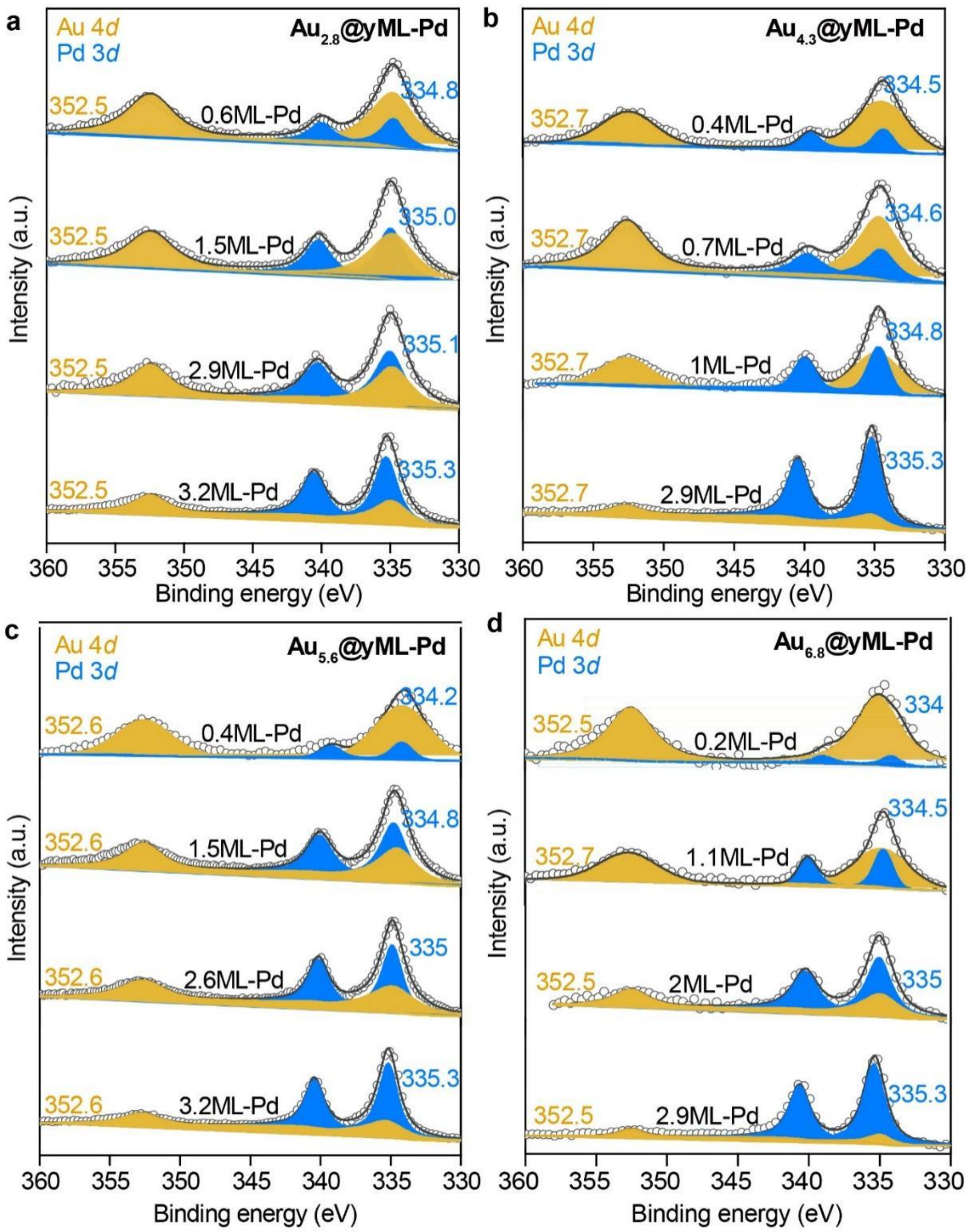

Supplementary Figure 19 | XPS spectra of Au_x@yML-Pd catalysts in Pd 3*d* and Au 4*d* region. a, $Au_{2.8}$@*y*ML-Pd. **b,** $Au_{4.3}$@*y*ML-Pd. **c,** $Au_{5.6}$@*y*ML-Pd. **d,** $Au_{6.8}$@*y*ML-Pd.

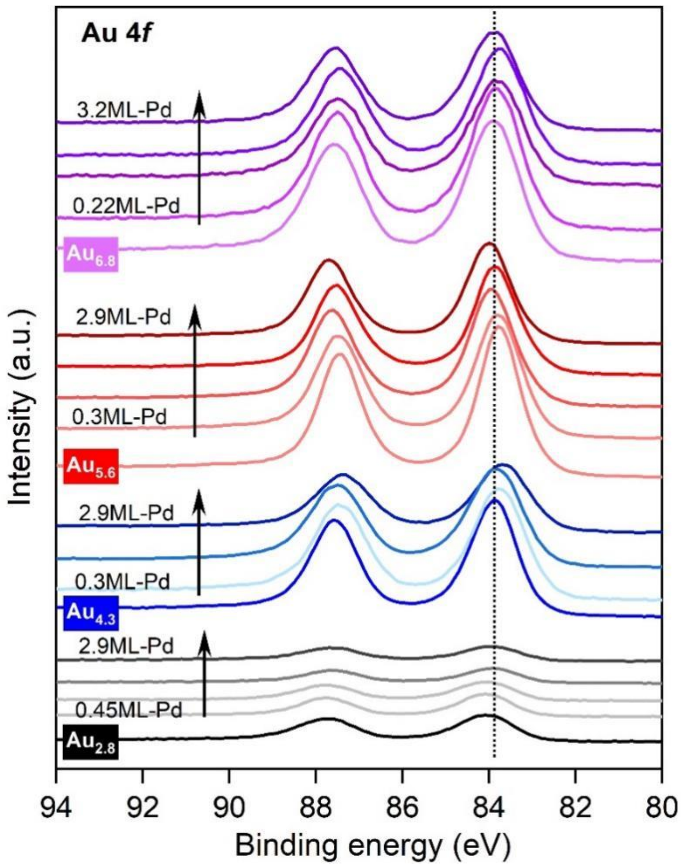

Supplementary Figure 20 | XPS spectra of Au_x@yML-Pd catalysts in Au 4*f* region.

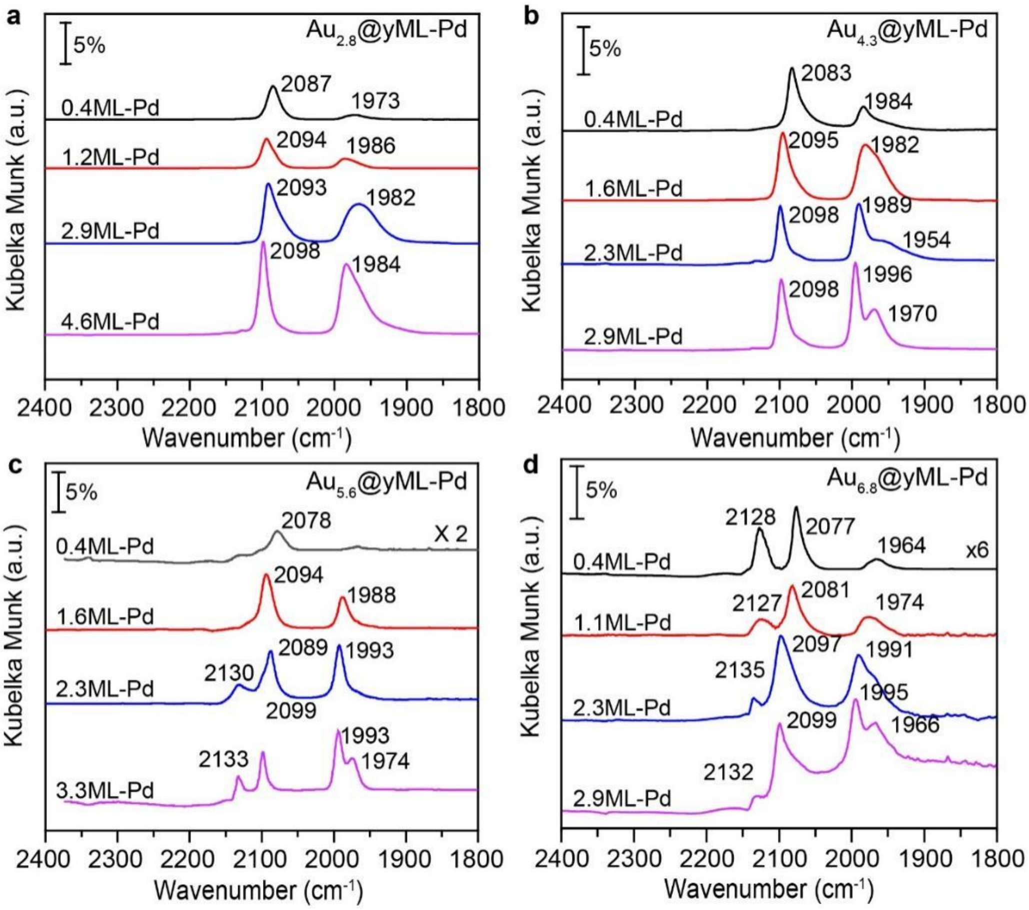

Supplementary Figure 21 | CO DRIFT spectra of Au$_x$@yML-Pd catalysts at the CO saturation coverage. a, Au$_{2.8}$@*y*ML-Pd. **b,** Au$_{4.3}$@*y*ML-Pd. **c,** Au$_{5.6}$@*y*ML-Pd. **d,** Au$_{6.8}$@*y*ML-Pd.

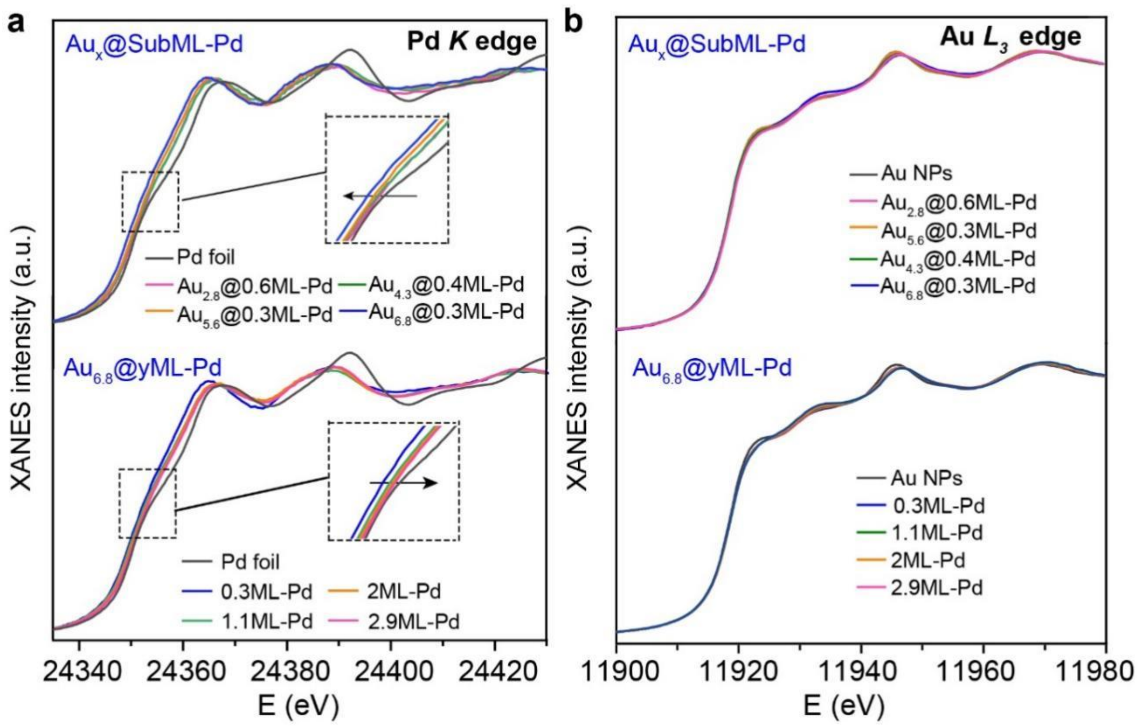

Supplementary Figure 22 | XAFS measurements of Au@Pd core-shell catalysts. a, *in situ* XANE spectra of Au_x@SubML-Pd and $Au_{6.8}$@yML-Pd catalysts at the Pd *K* edge. The insets show the enlargement of the Pd absorption edges of these catalysts. **b**, *Ex situ* XANE spectra of Au_x@SubML-Pd and $Au_{6.8}$@yML-Pd catalysts at the Au L_3 edge.

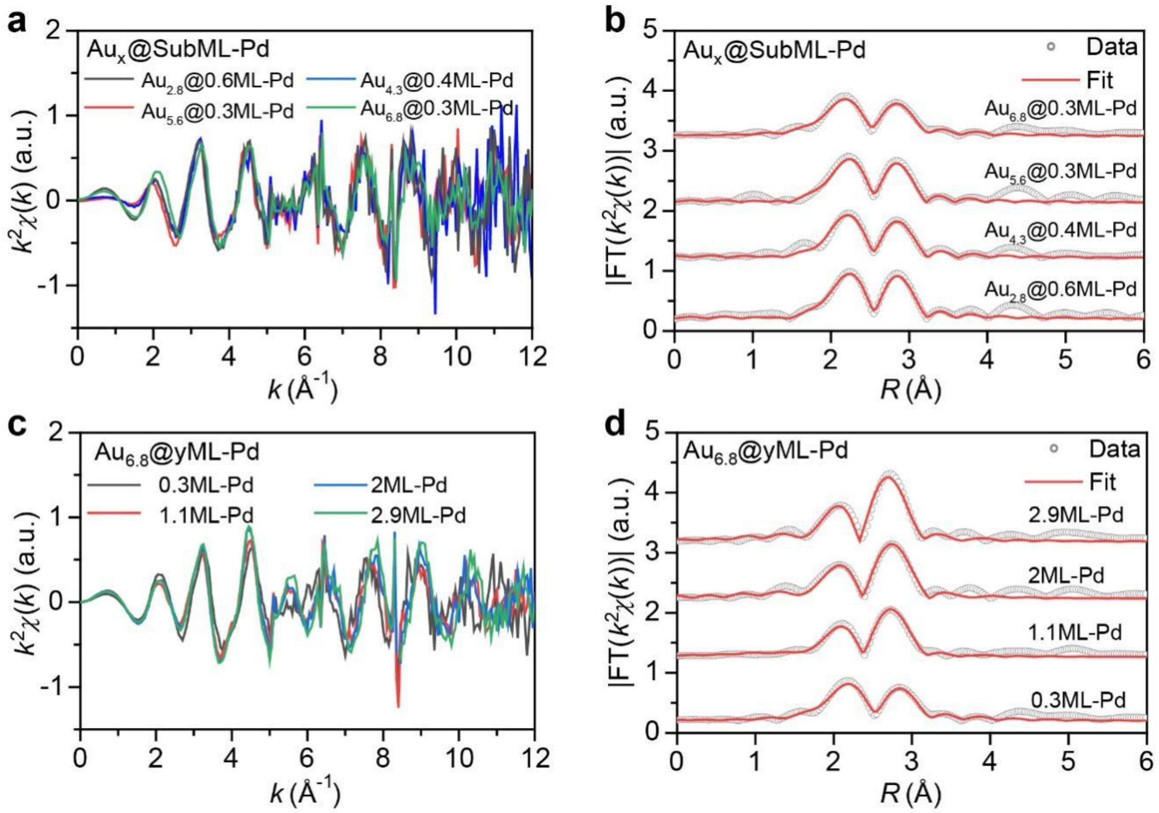

Supplementary Figure 23 | *In-situ* EXAFS spectra of Au@Pd core-shell catalysts at Pd *K*-edge. a, k^2-weighted EXAFS oscillations [$k^2\chi(k)$] of Au$_x$@SubML-Pd catalysts. **b,** the corresponding k^2-weighted Fourier transforms EXAFS spectra, as well as the fitting curves of Au$_x$@SubML-Pd catalysts. **c,** k^2-weighted EXAFS oscillations [$k^2\chi(k)$] of Au$_{6.8}$@yML-Pd catalysts. **d,** the corresponding k^2-weighted Fourier transforms EXAFS spectra, as well as the fitting curves of Au$_{6.8}$@yML-Pd catalysts. Here $\chi(k)$ denotes the amplitude of the EXAFS oscillations as a function of photoelectron wavenumber k, and R represents the distance between the absorbing atom and neighboring scattered atoms, without correcting for scattering phase shift.

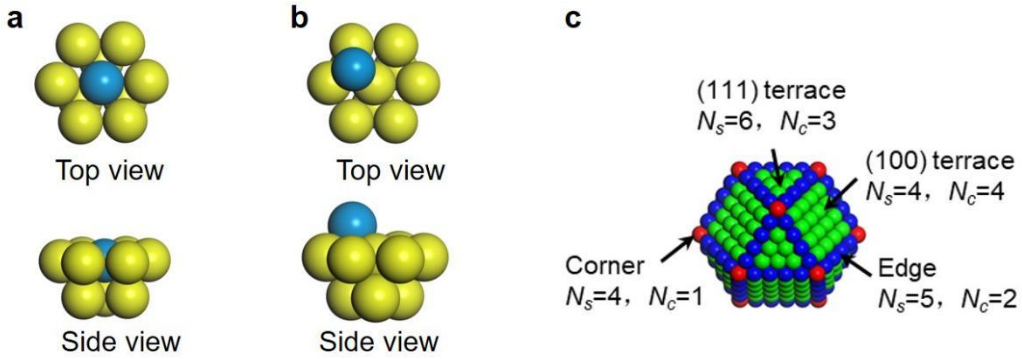

d

Sample	Adatom alloy	Surface alloy	$Au_{6.8}$@1ML-Pd	$Au_{6.8}$@2ML-Pd	$Au_{6.8}$@3ML-Pd
CN(Pd-Au)	3	9	3	1.4	0.9
CN(Pd-Pd)	0	0	6	9	10
CN(Pd_{Total})	3	9	9	10.4	10.9
CN(Au-Pd)			0.7	0.7	0.7
CN(Au-Au)			11.3	11.3	11.3
CN(Au_{Total})			12	12	12

Supplementary Figure 24 | Estimation of CNs in $Au_{6.8}$@yML-Pd core-shell catalysts according to the cubic-octahedral cluster model. a, Top and side views of the model of a surface alloy structure. **b**, Top and side views of the model of an adatom alloy structure. **c**, CNs of different surface atoms in a cubic-octahedral cluster. **d**, Estimated CNs of Pd-Au, Pd-Pd, Au-Pd and Au-Au coordinations in $Au_{6.8}$@1ML-Pd, $Au_{6.8}$@2ML-Pd, and $Au_{6.8}$@3ML-Pd according to the cluster model in **c**.

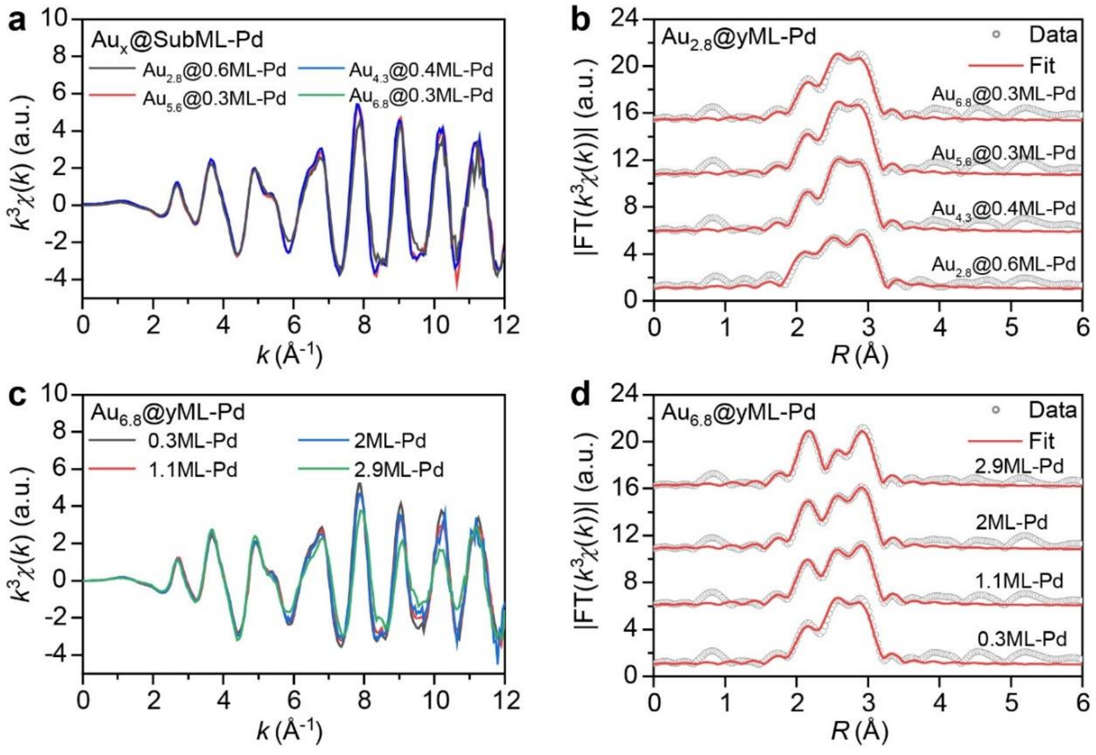

Supplementary Figure 25 | EXAFS spectra of Au_x@PdyML-Pd core-shell catalysts at Au L_3 edge. a, k^3-weighted EXAFS oscillations [$k^3\chi(k)$] of Au_x@SubML-Pd catalysts. **b,** the corresponding k^3-weighted Fourier transforms EXAFS spectra, as well as the fitting curves of Au_x@SubML-Pd catalysts. **c,** k^3-weighted EXAFS oscillations [$k^3\chi(k)$] of $Au_{6.8}$@yML-Pd catalysts. **d,** the corresponding k^3-weighted Fourier transforms EXAFS spectra, as well as the fitting curves of $Au_{6.8}$@yML-Pd catalysts.

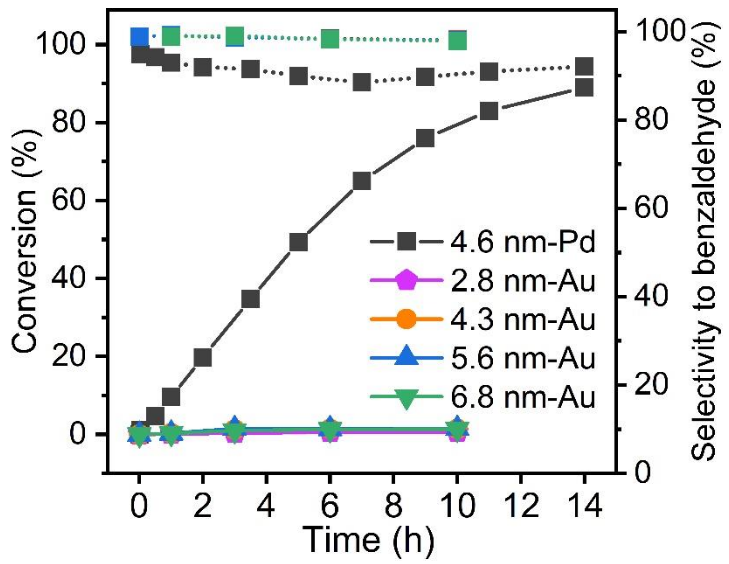

Supplementary Figure 26 | Catalytic performance of 4.6 nm-Pd catalyst and *x* nm-Au catalysts in solvent-free BzOH oxidation. Reaction conditions: benzyl alcohol, 5 mL; catalyst, 15 and 50 mg for monometallic Pd and Au catalysts, respectively; O_2, 15 mL min^{-1}; stirring speed, 1250 rpm; temperature, 90 ℃.

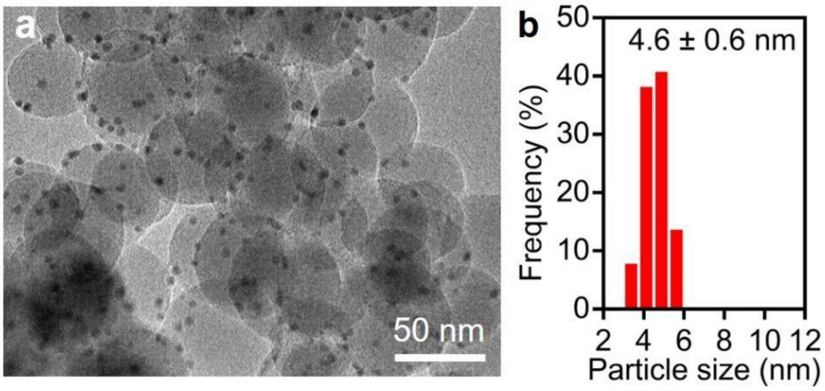

Supplementary Figure 27 | Morphology of the 4.6 nm-Pd catalyst. a, A representative TEM image. **b**, The corresponding Pd particle size distribution.

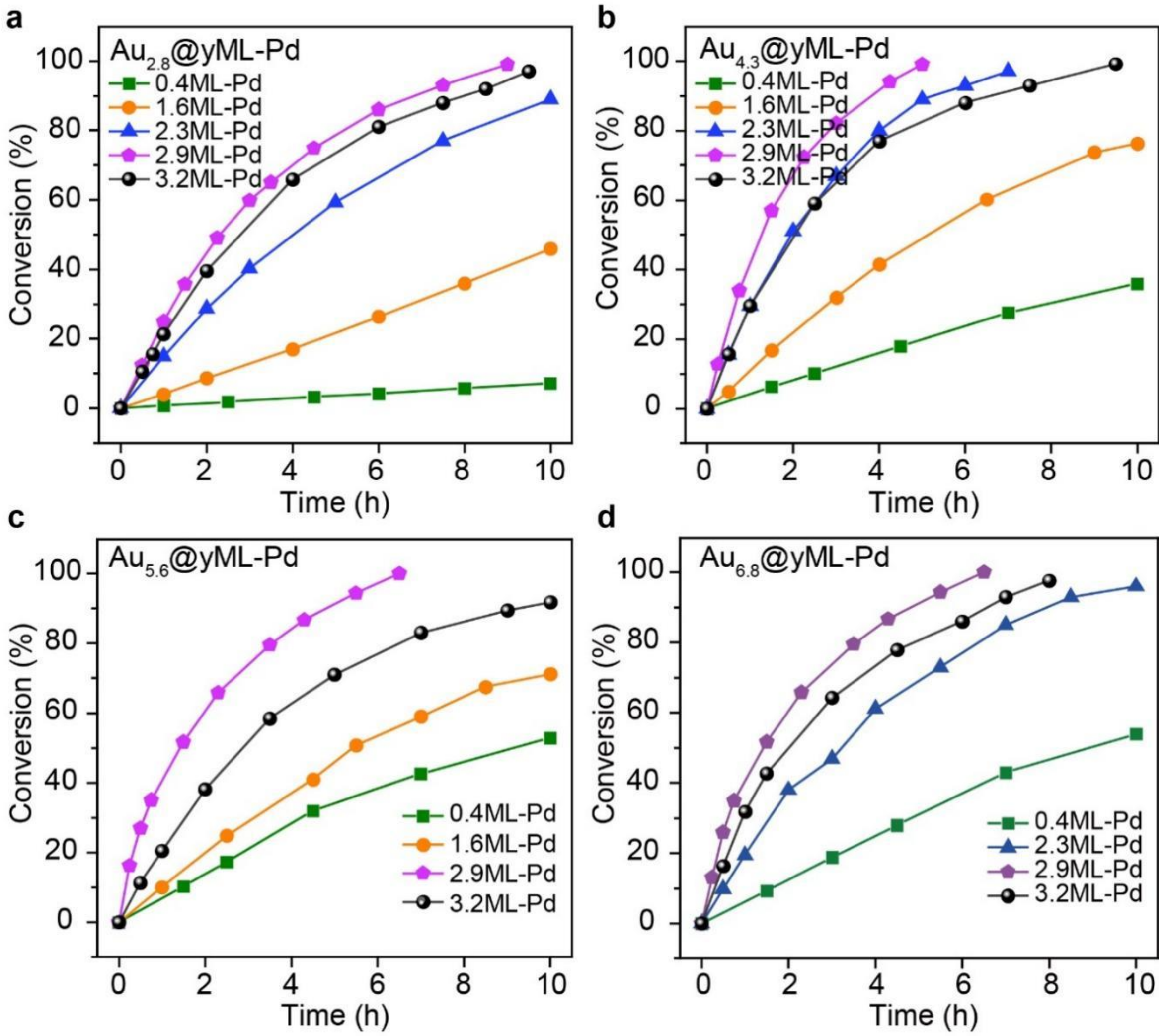

Supplementary Figure 28 | Conversion of BzOH over Au@Pd core-shell catalysts as a function of reaction time. a, $Au_{2.8}$@*y*ML-Pd. **b,** $Au_{4.3}$@*y*ML-Pd. **c,** $Au_{5.6}$@*y*ML-Pd. **d,** $Au_{6.8}$@*y*ML-Pd. Reaction conditions: BzOH, 5 mL; catalyst, 15 mg, except 50 mg for Au_x@0.4ML-Pd; O_2, 15 mL min^{-1}; stirring speed, 1250 rpm; temperature, 90 ℃.

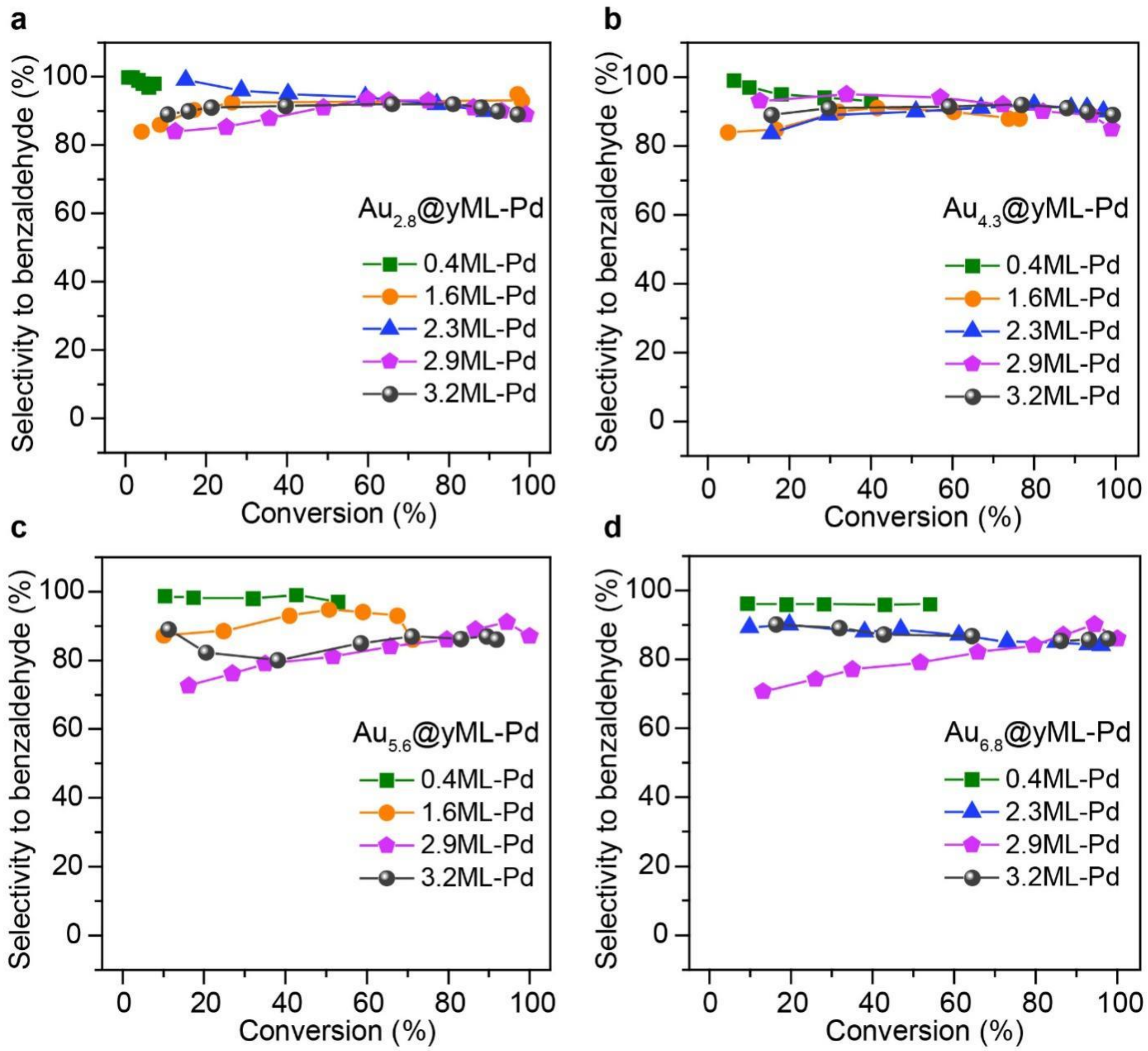

Supplementary Figure 29 | Selectivity of benzaldehyde over Au@Pd core-shell catalysts as a function of conversion. a, $Au_{2.8}$@*y*ML-Pd. **b,** $Au_{4.3}$@*y*ML-Pd. **c,** $Au_{5.6}$@*y*ML-Pd. **d,** $Au_{6.8}$@*y*ML-Pd. Reaction conditions: BzOH, 5 mL; catalyst, 15 mg, except 50 mg for Au_x@0.4ML-Pd; O_2, 15 mL min^{-1}; stirring speed, 1250 rpm; temperature, 90 ℃.

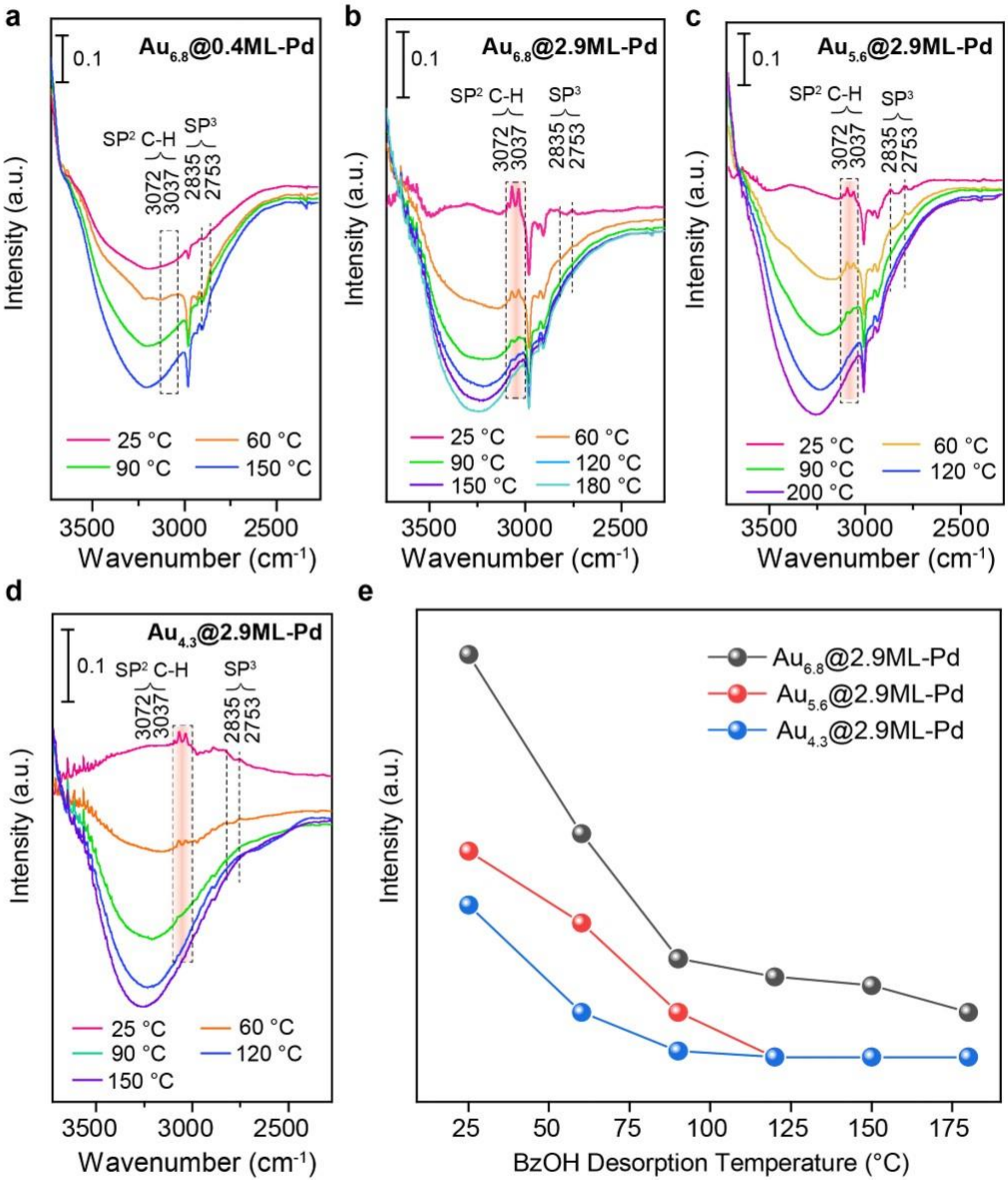

Supplementary Figure 30 | DRIFT spectra of adsorption of BzOH on various Au@Pd core shell catalysts at different temperatures. **a**, $Au_{6.8}$@0.4ML-Pd; **b**, $Au_{6.8}$@2.9ML-Pd; **c**, $Au_{5.6}$@2.9ML-Pd; and **d**, $Au_{4.3}$@2.9ML-Pd. **e**, Evolutions of the intensity of characteristic peak of C-H stretching at 3072 cm^{-1} (highlighted in red) as a function of temperature.

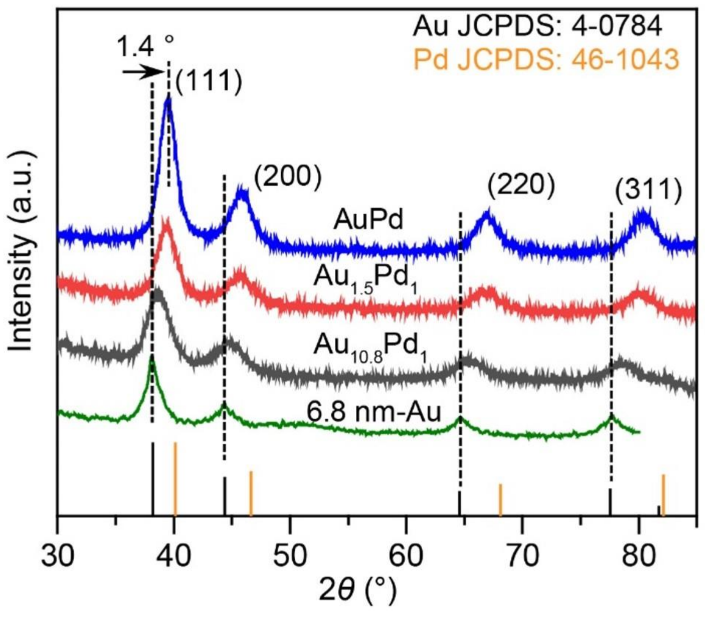

Supplementary Figure 31 | XRD spectra of Au_xPd_y alloy catalysts.

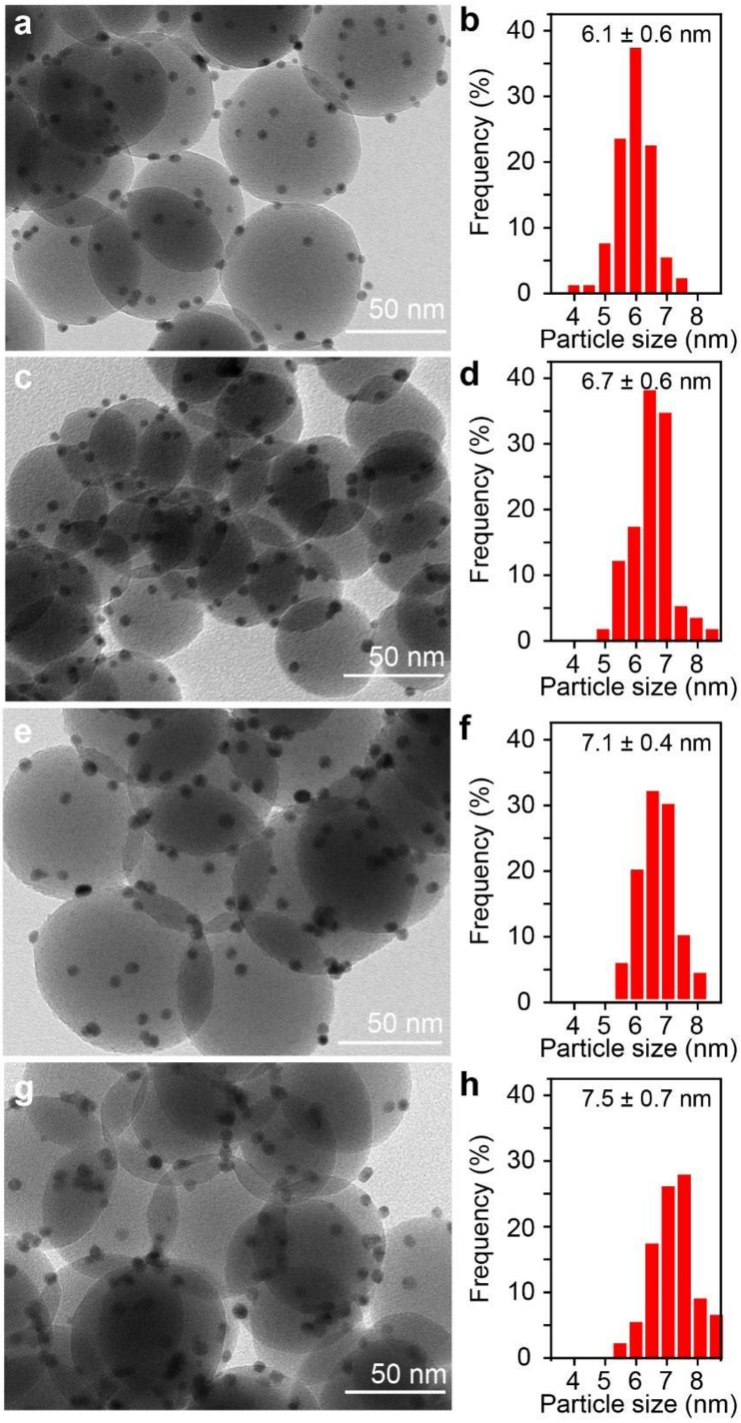

Supplementary Figure 32 | Representative TEM images of Au_xPd_y alloy catalysts and their corresponding histograms of particle size distribution. **a**,**b**, $Au_{10.8}Pd_1$; **c**,**d**, $Au_{1.5}Pd_1$; **e**,**f**, $Au_{1.2}Pd_1$; **g**,**h**, Au_1Pd_1.

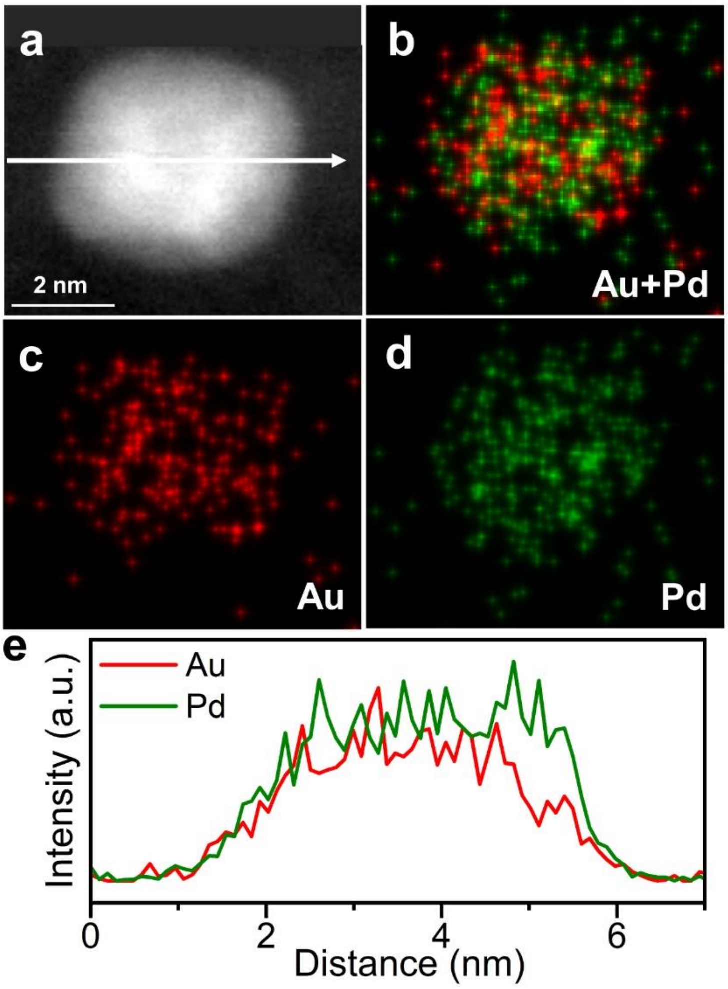

Supplementary Figure 33 | STEM EDS mapping and line profiles of an $Au_{1.2}Pd_1$ alloy particle. a, STEM image and the corresponding EDS mapping of constructed Au+Pd (**b**), Au Lα1 (**c**) and Pd Lα1 (**d**) signals. **e**, EELS line intensity profiles along the white arrow in **a**.

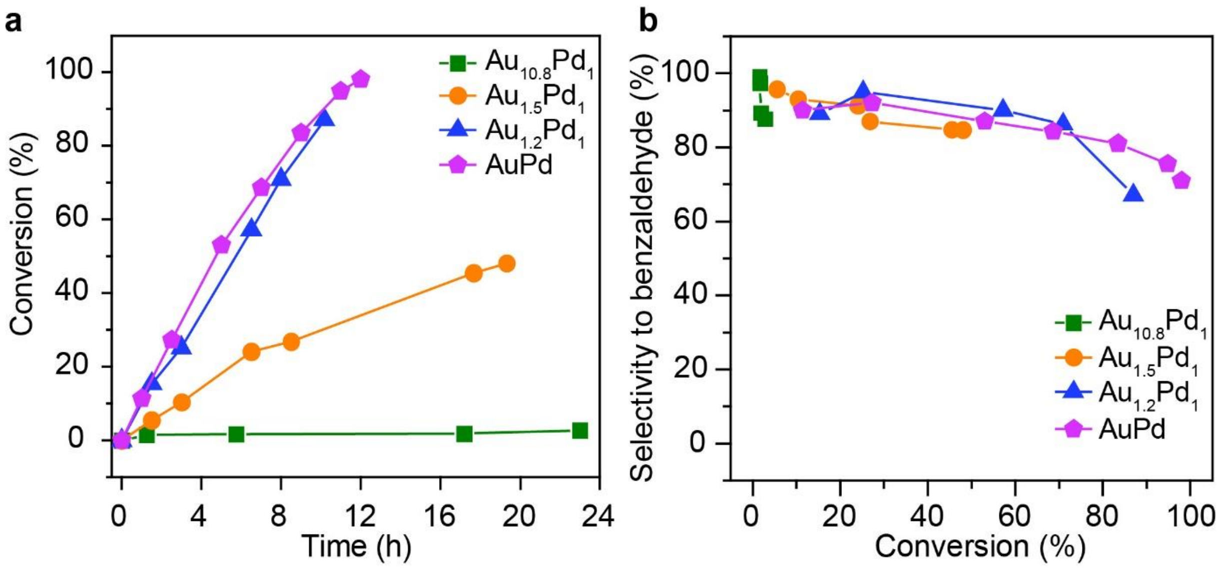

Supplementary Figure 34 | Catalytic performance of Au_xPd_y alloy catalysts in selective aerobic oxidation of BzOH. a, Conversion of BzOH as a function of reaction time. **b,** The benzaldehyde selectivity as a function of BzOH conversion. Reaction conditions: BzOH, 5 mL; catalyst, 15 mg; atmospheric pressure; temperature, 90 ℃.

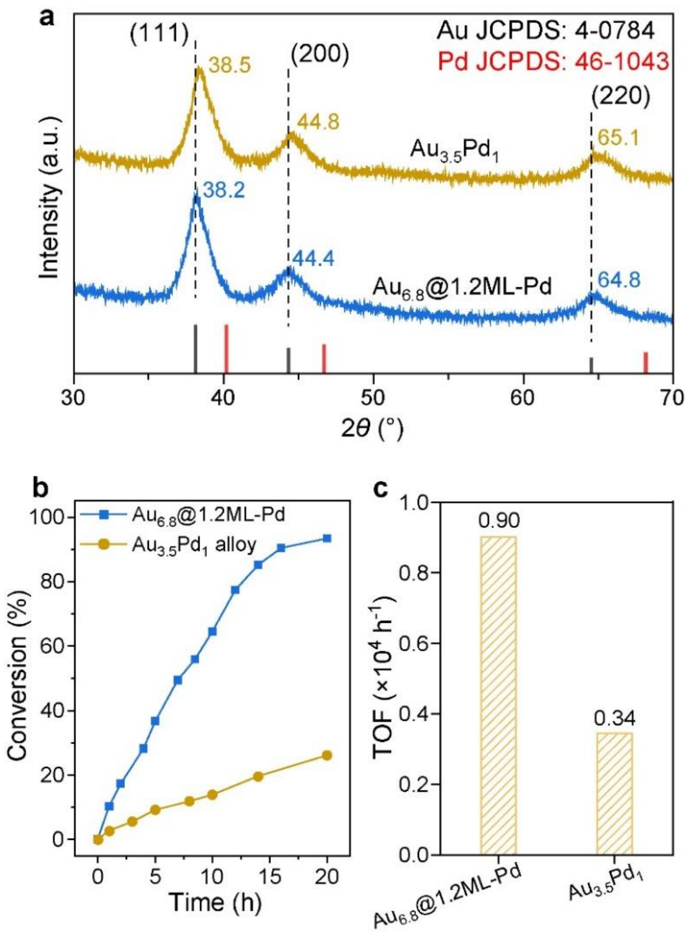

Supplementary Figure 35 | Comparison of Au@Pd core-shell and AuPd alloy catalysts in aerobic oxidation of BzOH. a, XRD spectra of the $Au_{6.8}$@1.2ML-Pd core-shell catalyst before and after reducing at 250 °C for 4 h ($Au_{3.5}Pd_1$)**. b,** Conversion of BzOH as a function of reaction time over the $Au_{6.8}$@1.2ML-Pd and $Au_{3.5}Pd_1$ alloy catalysts**. c,** TOFs of these two catalysts. Reaction conditions: BzOH, 5 mL; catalyst, 15 mg; atmospheric pressure; temperature, 90 ℃.

Supplementary note:

To examine the influence of calcination temperature, we performed another control experiment over the $Au_{6.8}$@1.2ML-Pd core-shell catalyst by decreasing the reduction temperature from 550 to 250 ℃, but with an extended reduction time of 4 h to ensure the formation of AuPd alloy. The reduced sample was denoted as $Au_{3.5}Pd_1$ with an Au to Pd atomic ratio of 3.5 : 1. XRD showed that the diffraction peaks were located at 38.2, 44.4 and 64.8 ° on $Au_{6.8}$@1.2ML-Pd, assigned to Au (111), Au(200) and Au(220), respectively, confirming the core-shell structure (Supplementary Fig. 35a). After extended reduction at 250 ℃ for 4 h, these

peaks shifted considerably to 38.5, 44.8 and 65.1 ° on $Au_{3.5}Pd_1$, ambiguously confirming the alloy formation. In the reaction of solvent-free oxidation of BzOH, we showed that the activity of $Au_{3.5}Pd_1$ was only 0.34×10^4 h^{-1}, much smaller than that of $Au_{6.8}$@1.2ML-Pd (0.90×10^4 h^{-1}) (Supplementary Fig. 35b, c). This result clearly verifies that the large activity decline on AuPd alloy catalysts compared with those Au@Pd core-shell catalysts (Fig. 5d) is solely attributed to the change in catalyst structure instead of metal-support interactions.

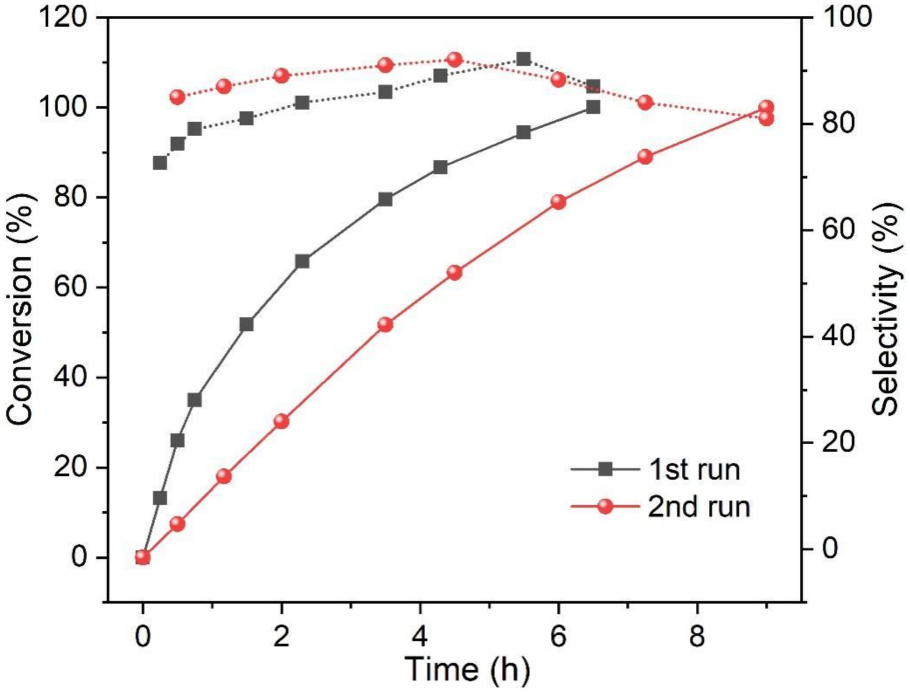

Supplementary Figure 36 | Recyclability test of $Au_{6.8}$@2.9ML-Pd catalysts. Reaction conditions: BzOH, 5 mL; catalyst, 15 mg; atmospheric pressure; temperature, 90 ℃.

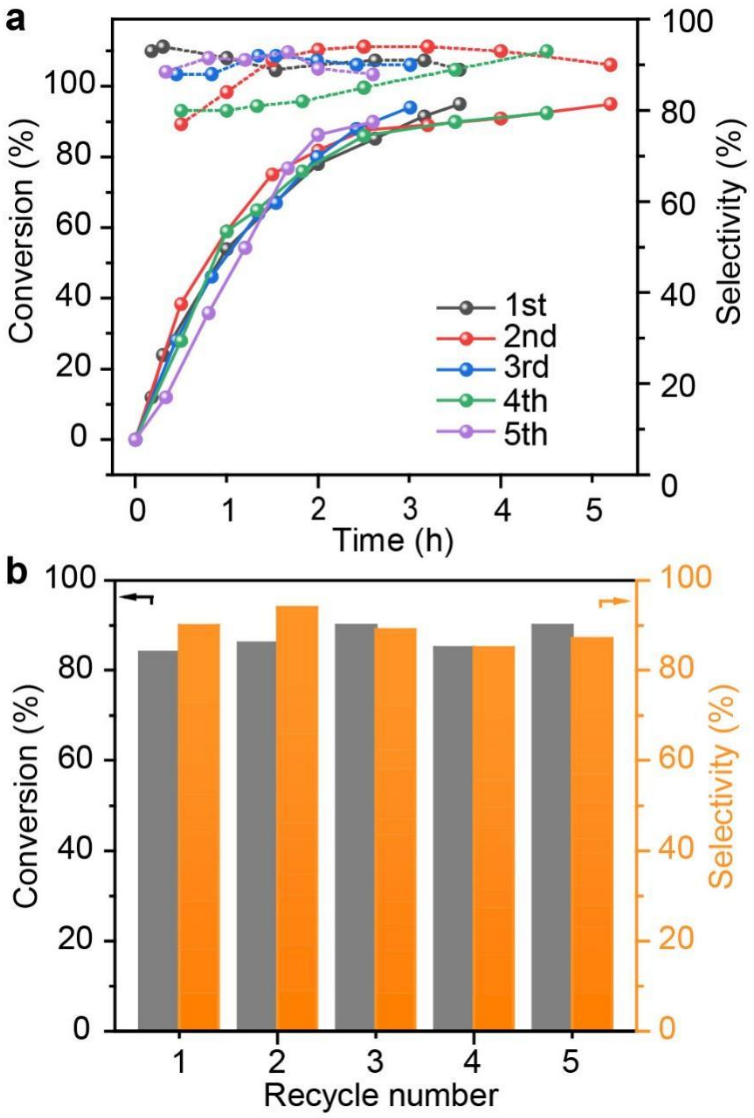

Supplementary Figure 37 | Recyclability test of $Au_{6.5}$@2.9ML-Pd/SBA-15 catalyst in selective aerobic oxidation of BzOH. a, BzOH conversion and benzaldehyde selectivity as a function of reaction time in different recycles. **b,** Comparison BzOH conversion and benzaldehyde selectivity after 2.5 h reaction. Reaction conditions: BzOH, 5 mL; catalyst, 15 mg; atmospheric pressure; temperature, 90 ℃.

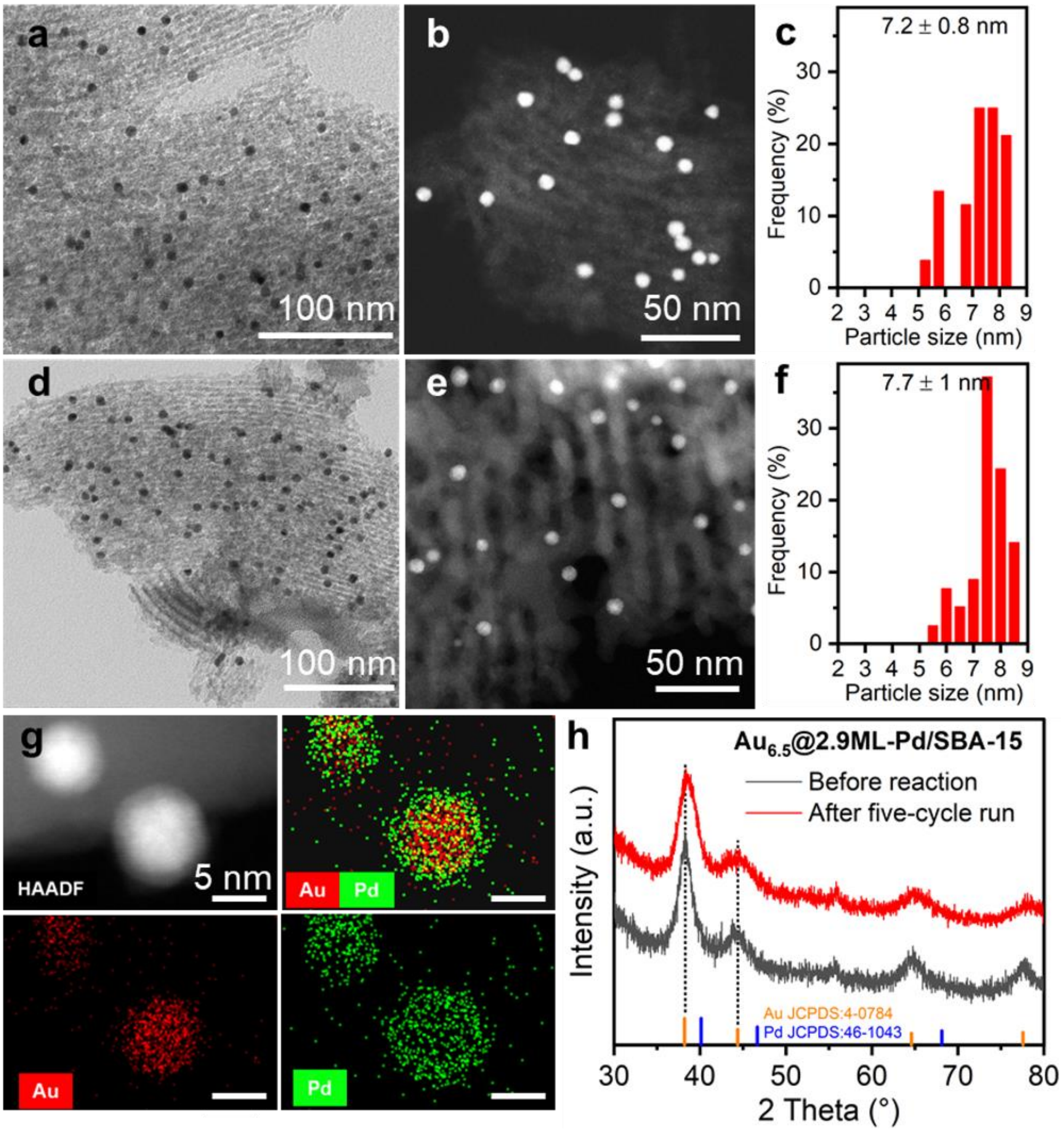

Supplementary Figure 38 | Morphology of fresh and used Au$_{6.5}$@2.9ML-Pd/SBA-15 catalyst. a-c, TEM and HAADF-STEM images, as well as the particle size distribution of the fresh Au$_{6.5}$@2.9ML-Pd/SBA-15 catalyst**. d-f,** TEM and HAADF-STEM images as well as the particle size distribution of the used Au$_{6.5}$@2.9ML-Pd/SBA-15 catalyst after 5-cycle-run recyclability test. **g**, A STEM image and the corresponding EDS elemental mapping (Pd Lα1, Au Lα1 and constructed Pd + Au signals) of the used Au$_{6.5}$@2.9ML-Pd/SBA-15 catalyst after 5-cycle-run recyclability test. **h**, Comparison of XRD patterns of Au$_{6.5}$@2.9ML-Pd/SBA-15 before and after 5-cycle-run recyclability test.

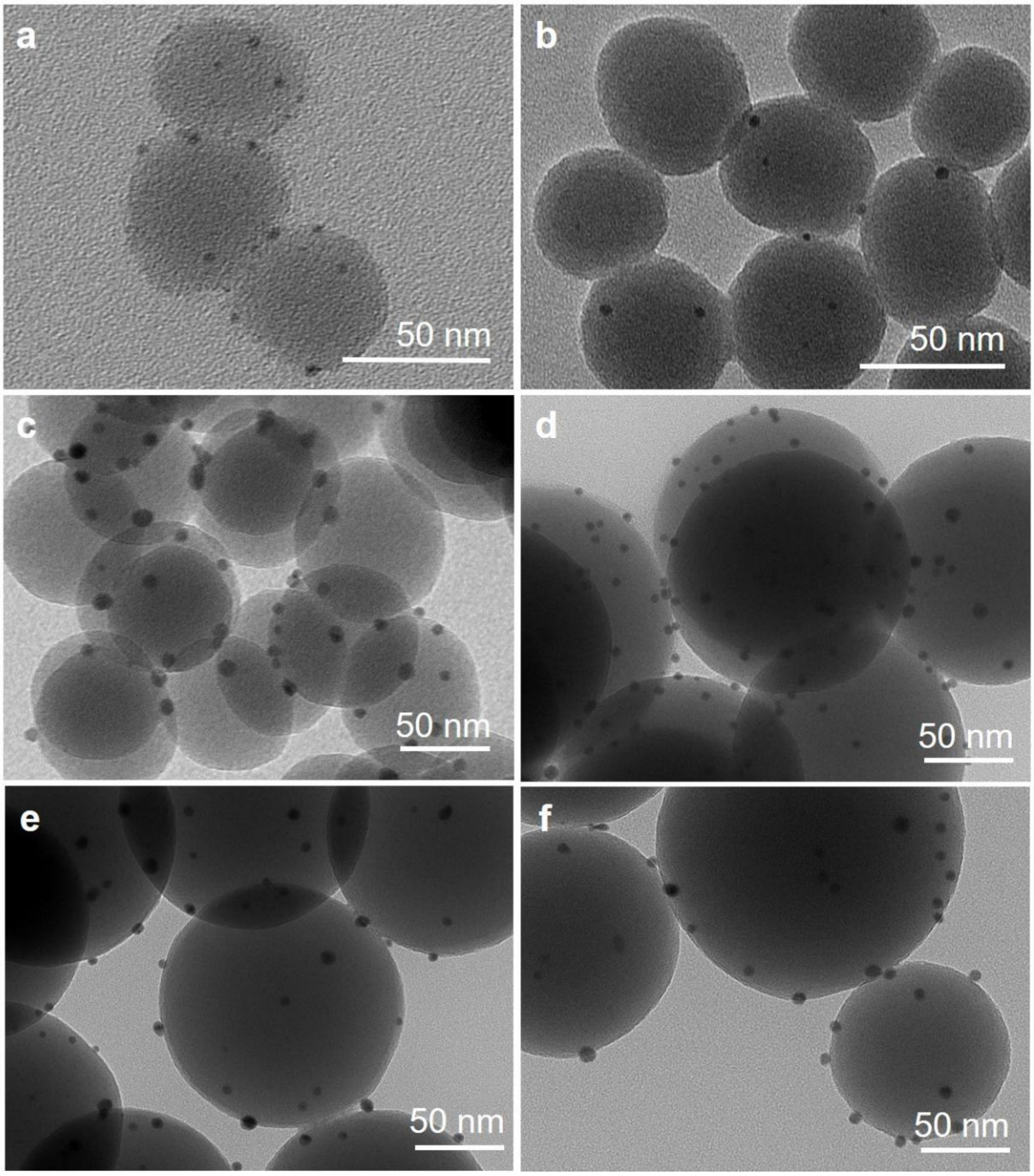

Supplementary Figure 39 | Representative TEM images of Au$_x$@yML-Pt core-shell catalysts. **a**, Au$_{3.1}$@1ML-Pt; **b**, Au$_{4.2}$@1ML-Pt; **c**, Au$_{6.5}$@1ML-Pt; **d**, Au$_{5.3}$@0.5ML-Pt, **e,** Au$_{5.3}$@1ML-Pt and **f**, Au$_{5.3}$@2ML-Pt.

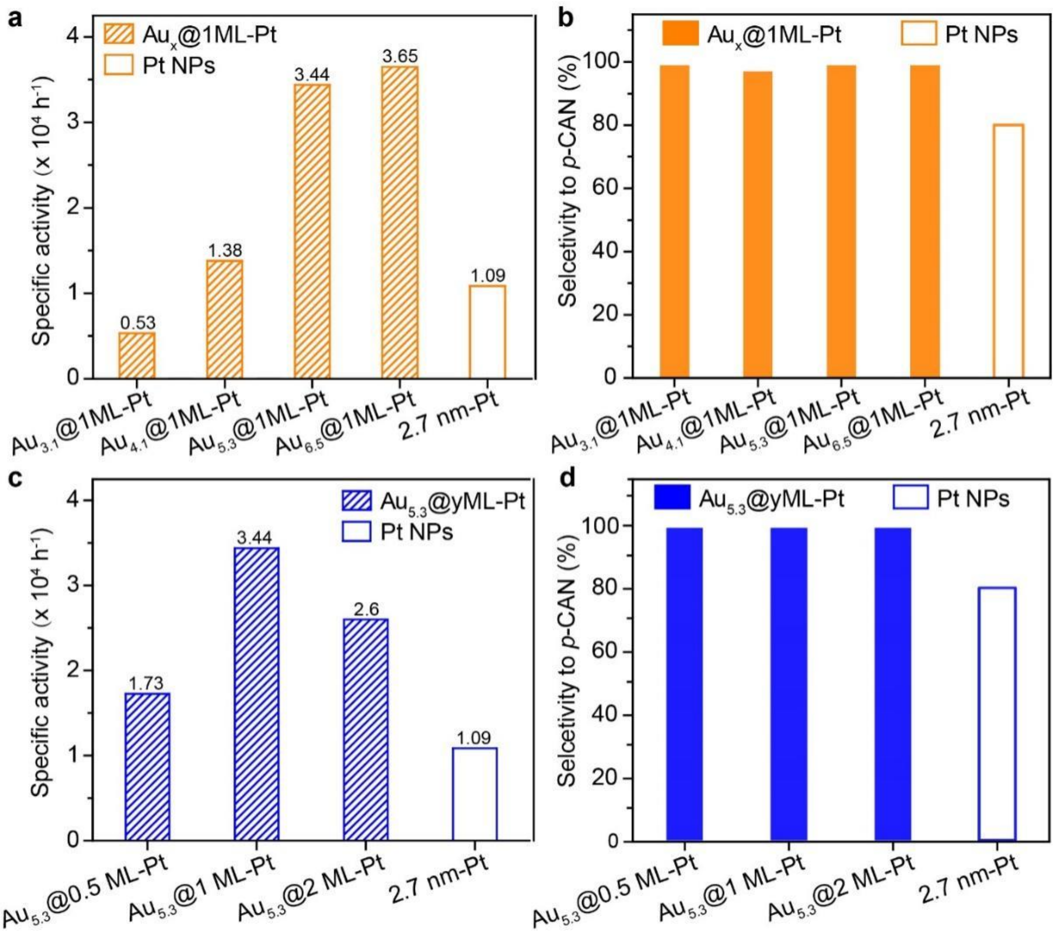

Supplementary Figure 40 | Comparison of catalytic performance of Pt based catalysts in chemoselective hydrogenation of *para*-chloronitrobenzene. Comparison of specific activity (**a**) and selectivity to *para*-chloroaniline (*p*-CAN) (**b**) of the Au_x@1ML-Pt core-shell bimetallic catalysts with a Pt shell thickness of 1ML and different Au core sizes. Comparison of specific activity (**c**) and selectivity to *para*-chloroaniline (*p*-CAN) (**d**) of the $Au_{5.3}$@*x*ML-Pt core-shell bimetallic catalysts with an Au core size of 5.3 nm and different Pt shell thickness. The 2.7 nm-Pt catalysts was also shown in (**a-d**) as a reference for comparison. Reaction conditions: solvent, ethanol, 40 mL; H_2 pressure, 0.3 MPa; temperature, 65 ℃; *p*-CNB, 8 mmol; and Pt : substrate mole ratio, 1:7800 for the catalysts used.

Supplementary Table 1 | The calculated (111) facet distance and edge length of different Au cluster models.

Au cluster	Au cluster size[a] (nm)	(111) interplane spacing (Å)		Contraction[b] (%)	(111) facet edge length (Å)		Contraction (%)
		relaxed value	bulk truncated value		relaxed value	bulk truncated value	
Au_{55}	1.3	9.0	9.5	5.26	5.54	5.83	4.97
Au_{147}	1.7	13.7	14.3	4.20	8.39	8.74	4.00
Au_{309}	2.2	18.3	19.0	3.68	11.21	11.66	3.86
Au_{561}	2.7	22.9	23.8	3.78	14.05	14.57	3.57
Au_{923}	3.2	27.6	28.6	3.50	16.91	17.49	3.32

[a] Determined on the literature[1].
[b] Contraction (%) = (bulk truncated value – relaxed value)/(bulk truncated value) ×100%

Supplementary Table 2 | The (111) interlayer distance measured from HAADF-STEM images and the corresponding contractions compared to Au crystals.

Au particle size (nm)	(111) interlayer distance (nm)	Contraction[a] (%)
2.4	0.226	4.24
2.9	0.227	3.81
3.1	0.230	2.54
3.5	0.232	1.69
3.7	0.234	0.85
5.3	0.235	0.42
Au-bulk	0.236[b]	0

[a] Contraction (%) = (Au-bulk (111) – Au-*x*nm (111))/(Au-bulk (111)) ×100%.
[b] Data from PDF#04-0784.

Supplementary Table 3 │ Calculated *d*-band centers (all in eV) for topmost surface metal atom, subsurface metal atom, and their sum for various clean surfaces considered.

Surface	$\varepsilon_{d\text{-surf}}$	$\varepsilon_{d\text{-subsurf}}$	$\varepsilon_{d\text{-sum}}$
3ML-Au(111)/1ML-Pd(111)[a]	-1.63	-3.59	-5.22
3ML-Au(111)/2ML-Pd(111)	-1.64	-2.01	-3.65
3ML-Au(111)/3ML-Pd(111)	-1.75	-2.22	-3.97
3ML-Au(111)/4ML-Pd(111)	-1.76	-2.27	-4.03
3ML-Au(111)/5ML-Pd(111)	-1.74	-2.26	-4.00
3ML-Au(111)/6ML-Pd(111)	-1.74	-2.25	-3.98
Stretched 4ML-Pd(111)[b]	-1.78	-2.33	-4.11
Stretched 5ML-Pd(111)	-1.74	-2.27	-4.01
Stretched 6ML-Pd(111)	-1.71	-2.22	-3.93
Stretched 7ML-Pd(111)	-1.71	-2.19	-3.90
4ML-Pd(111) [c]	-1.96	-2.37	-4.33

[a] Bottom two Au(111) layers fixed at bulk position, the rest of layers fully relaxed. Equilibrium lattice constant of Au is used.

[b] Bottom two Pd(111) layers fixed, the rest of Pd(111) layers fully relaxed. Equilibrium lattice constant of Au is used.

[c] Bottom two Pd(111) layers fixed, the rest of Pd(111) layers fully relaxed. Equilibrium lattice constant of Pd is used.

Supplementary Table 4 | Metal loadings and dispersions of Pd-based catalysts.

Catalyst	Pd ALD cycles	Metal loadings (wt%)		Pd/Au ratio	Dispersion[a] (%)
		Pd	Au		
$Au_{2.8}$@0.6ML-Pd	1	0.1	0.7	1:3.78	100
$Au_{2.8}$@1.6ML-Pd	4	0.41	0.7	1.16:1	69
$Au_{2.8}$@2.3ML-Pd	8	0.71	0.7	1.87:1	53
$Au_{2.8}$@2.9ML-Pd	10	0.96	0.7	2.53:1	45
$Au_{2.8}$@3.2ML-Pd	11	1.12	0.7	2.91:1	40
$Au_{4.3}$@0.4ML-Pd	1	0.24	2.8	1:6.3	100
$Au_{4.3}$@1.6ML-Pd	5	1.03	2.8	1:1.47	51
$Au_{4.3}$@2.3ML-Pd	15	1.6	2.8	1.05:1	30
$Au_{4.3}$@2.9ML-Pd	18	2.13	2.8	1.41:1	24
$Au_{4.3}$@3.2ML-Pd	20	2.44	2.8	1.61:1	19
$Au_{5.6}$@0.3ML-Pd	1	0.17	2.8	1:8.9	94.2
$Au_{5.6}$@1.6ML-Pd	7	0.81	2.8	1:1.87	34
$Au_{5.6}$@2.3ML-Pd	11	1.24	2.8	1:1.22	26
$Au_{5.6}$@2.9ML-Pd	13	1.63	2.8	1.08:1	20
$Au_{5.6}$@3.2ML-Pd	15	1.85	2.8	1.22:1	13
$Au_{6.8}$@0.3ML-Pd	1	0.14	2.8	1:10.8	82
$Au_{6.8}$@1.6ML-Pd	10	0.66	2.8	1:2.29	30
$Au_{6.8}$@2.3ML-Pd	13	1	2.8	1:1.51	26
$Au_{6.8}$@2.9ML-Pd	20	1.32	2.8	1:1.15	19
$Au_{6.8}$@3.2ML-Pd	23	1.49	2.8	1:1.01	15
$Au_{6.5}$@2.9ML-Pd/SBA-15	23	1.32	2.7	1:1	27
$Au_{10.8}Pd_1$	1	0.14	2.8	1:10.8	23.7
$Au_{1.5}Pd_1$	13	1	2.8	1:1.51	19
$Au_{1.2}Pd_1$	20	1.32	2.8	1:1.15	12
Au_1Pd_1	23	1.49	2.8	1:1.01	11
2.8 nm-Au	-	-	0.7	-	-
4.3 nm-Au	-	-	2.8	-	-
5.6 nm-Au	-	-	2.8	-	-
6.8 nm-Au	-	-	2.8	-	-
4.6 nm-Pd	-	3.5	-	-	28

[a] Pd dispersion determined by CO pulse chemisorption.

Supplementary Table 5 | The change of the number of metal atom shells as the size of Au NPs according to the magic cluster model.

Shell number	Shell atoms	Total atoms	Calculated magic diameter (nm)
5	252	561	2.97
6	362	923	3.51
7	492	1415	4.05
8	642	2057	4.59
9	812	2869	5.13
10	1002	3871	5.67
11	1212	5083	6.21
12	1442	6525	6.75
13	1692	8217	7.29

Typically, for 6.8 nm Au NPs contains approximate 1442 shell atoms and 5083 core atoms according to cluster model, the molar ratio of shell/core was 0.284. With the Pd loadings of 1.32 wt% and Au loadings of 2.8 wt% in $Au_{6.8}$@2.9ML-Pd catalysts, the molar ratio of Pd/Au was 0.87. Therefore, the coverage of Pd shells on 6.8 nm-Au NPs was 2.9 monolayers. All the coverages of Pd shells of Au@Pd catalysts were calculated in a similar way.

Supplementary Table 6 | Structural parameters from quantitative EXAFS curve-fittings using the ARTEMIS module of IFEFFIT at the Pd *K*-edge. For comparison, the interatomic distances and coordination numbers for the references (Pd foil) calculated from their crystallographic structures are also listed.

Sample	Path	CNs	*R* (Å)	σ^2 (10^{-3}Å^2)	ΔE_0 (eV)	R-factor
Pd foil	Pd-Pd	12	2.74±0.01	4.9±0.7	4.5±0.6	0.006
$Au_{2.8}$@0.6ML-Pd	Pd-Pd	1.3±0.5	2.76±0.01	5.0±1.9	1.5±0.5	0.006
	Pd-Au	7.2±0.5	2.79±0.02	4.8±1.2	1.5±0.5	
$Au_{4.3}$@0.4ML-Pd	Pd-Pd	0.9±0.7	2.77±0.04	10.3±3.9	3.1±5.8	0.020
	Pd-Au	8.1±0.8	2.79±0.02	7.4±0.8	1.5±0.8	
$Au_{5.6}$@0.3ML-Pd	Pd-Pd	0.8±0.6	2.77±0.06	6.1±2.5	1.2±1.0	0.016
	Pd-Au	8.2±0.4	2.80±0.01	7.0±0.9	1.2±1.0	
$Au_{6.8}$@0.3ML-Pd	Pd-Pd	0.9±0.3	2.77±0.01	7.2±2.2	1.9±0.7	0.018
	Pd-Au	8.1±0.6	2.80±0.02	8.4±0.6	1.9±0.7	
$Au_{6.8}$@1.1ML-Pd	Pd-Pd	6.0±0.4	2.76±0.03	10.7±0.9	0.6±0.5	0.020
	Pd-Au	3.5±0.5	2.80±0.02	4.0±1.0	4.3±0.9	
$Au_{6.8}$@2ML-Pd	Pd-Pd	7.9±1.2	2.77±0.03	12.6±1.1	0.4±0.6	0.023
	Pd-Au	3.3±0.4	2.79±0.02	3.1±1.3	5.7±1.1	
$Au_{6.8}$@2.9ML-Pd	Pd-Pd	9.1±0.6	2.77±0.01	11.7±2.1	0.8±0.6	0.026
	Pd-Au	2.5±0.7	2.78±0.02	2.8±1.2	6.6±2.3	
$Au_{5.6}$@3.2ML-Pd	Pd-Pd	9.0±0.5	2.77±0.02	11.3±1.0	1.5±0.6	0.035
	Pd-Au	2.1±0.3	2.79±0.04	2.2±0.7	7.4±2.0	
$Au_{4.3}$@2.9ML-Pd	Pd-Pd	8.5±1.1	2.76±0.03	12.7±1.1	2.4±0.1	0.016
	Pd-Au	2.1±0.4	2.78±0.02	7.4±2.6	6.2±1.2	

Supplementary Table 7 | Structural parameters at the Au L_3-edge extracted from quantitative EXAFS curve-fittings using the ARTEMIS module of IFEFFIT. For comparison, the interatomic distances and coordination numbers for the references (Pd foil) calculated from their crystallographic structures are also listed.

Sample	Path	CNs	R (Å)	σ^2 (10^{-3}Å^2)	ΔE_0 (eV)	R-factor
Au foil	Au-Au	12	2.86±0.01	8.3±0.3	4.8±0.3	0.004
$Au_{2.8}$@0.6ML-Pd	Au-Au	10.3±0.2	2.84±0.01	9.1±0.5	3.9±0.7	0.016
	Au-Pd	0.6±0.5	2.79±0.03	5.4±4.9	3.9±0.7	
$Au_{4.3}$@0.4ML-Pd	Au-Au	10.9±0.5	2.85±0.01	9.1±0.3	4.7±0.4	0.004
$Au_{5.6}$@0.3ML-Pd	Au-Au	11.4±0.8	2.85±0.01	9.4±0.4	4.5±0.5	0.009
$Au_{6.8}$@0.3ML-Pd	Au-Au	11.3±0.8	2.84±0.04	9.0±0.5	5.1±0.5	0.013
$Au_{6.8}$@1.1ML-Pd	Au-Au	10.8±0.4	2.84±0.01	9.3±0.3	4.8±0.4	0.004
	Au-Pd	1.2±0.4	2.78±0.01	8.5±2.3	4.8±0.4	
$Au_{6.8}$@2ML-Pd	Au-Au	10.7±0.4	2.84±0.01	9.1±0.3	4.6±0.4	0.004
	Au-Pd	1.3±0.4	2.79±0.01	7.8±1.9	4.6±0.4	
$Au_{6.8}$@2.9ML-Pd	Au-Au	10.1±0.7	2.83±0.01	9.2±0.3	3.8±0.5	0.008
	Au-Pd	1.9±0.3	2.78±0.01	6.6±0.8	3.8±0.5	
$Au_{5.6}$@3.2ML-Pd	Au-Au	10.1±0.4	2.83±0.004	9.4±0.4	3.8±0.4	0.006
	Au-Pd	1.9±0.4	2.78±0.009	7.8±1.4	3.8±0.4	
$Au_{4.3}$@2.9ML-Pd	Au-Au	10.1±0.6	2.84±0.01	8.7±0.4	4.6±0.4	0.003
	Au-Pd	1.5±0.4	2.79±0.01	7.7±1.6	4.6±0.4	

Supplementary Table 8 | Comparison of catalyst performance of various Pd and AuPd catalysts in this work with those reported in literature in terms of activity in selective oxidation of benzyl alcohol.

Catalysts	Metal particle structure	Solvent	Reaction temperature (°C)	TOFs (h^{-1})	Ratio of TOF_{AuPd} to TOF_{Pd}	Notes
4.6 nm-Pd	4.6 nm NPs	solvent-free	90	3260	-	This work
$Au_{6.8}$@2.9ML-Pd/SiO_2	core-shell	solvent-free	90	68327	21.9	
$Au_{5.6}$@2.9ML-Pd/SiO_2	core-shell	solvent-free	90	65206	20.9	
$Au_{4.3}$@2.9ML-Pd/SiO_2	core-shell	solvent-free	90	32439	10.4	
$Au_{2.8}$@2.9ML-Pd/SiO_2	core-shell	solvent-free	90	18604	6	
Pd/SiO_2	4.3 nm NPs	solvent free	90	4320	-	Ref[2]
$Au_{4.3}$@2.9ML-Pd/SiO_2	core-shell	solvent-free	90	27600	9.2	
Pd/TiO_2	5~6 nm NPs	solvent free	100	2200	-	Ref[3]
AuPd/TiO_2	core-shell	solvent-free	100	6440	2.9	
Pd/Fe_2O_3@SiO_2	Nanoparticle	solvent-free	100	155	-	Ref[4]
AuPd/Fe_3O_4@SiO_2	core-shell	solvent-free	100	900	5.8	
Pd/SiO_2	3~4 nm NPs	solvent free	140	6027	-	Ref[5]
Pd/APS-S16	3~4 nm NPs	solvent free	140	7858	-	
1Au5Pd/APS-S16	core-shell	solvent-free	140	8667	1.1	
Pd/S15-p	5.3 nm NPs	solvent free	160	4780	-	Ref[6]
AuPd/SBA-15-p	core-shell	solvent-free	160	27000	2.25	
Pd/meso-C	Nanoparticle	H_2O	90	50	-	Ref[7]
$Au_{0.5}Pd_{0.5}$/meso-C	alloy	H_2O	90	370	7.5	
Pd/P12	Nanoparticle	solvent free	120	121	-	Ref[8]
$Au_{0.5}Pd_{0.5}$/TS-1	alloy	solvent-free	120	615	5.1	
PVP-stabilized Pd	3.9 nm NPs	H_2O+K_2CO	85	31.8	-	Ref[9]
PVP-stabilized $Au_{0.25}Pd_{0.75}$ NPs	alloy	$H_2O+K_2CO_3$	85	57.3	1.8	
Pd/C	3~4 nm NPs	H_2O	60	30	-	Ref[10]
$Au_{0.73}Pd_{0.27}$ sol/C	alloy	H_2O	60	160	5.3	
Pd/LDH	5 nm NPs	H_2O	80	70	-	Rref[11]
$Au_{0.5}Pd_{0.5}$/LDH	alloy	H_2O	80	140	2.0	
Pd/TiO_2	Nanoparticle	solvent-free	100	196	-	Ref[12]
$Au_{0.5}Pd_{0.5}$/TiO_2	alloy	solvent-free	100	310	1.58	
$Au_{0.5}Pd_{0.5}$/Y	alloy	solvent-free	100	280	0.82	Ref[13]

Supplementary Table 9 | Metal loadings and the Pt dispersions of Pt-based catalysts.

Catalyst	Pt ALD cycles	Metal loadings (wt%)		Pt dispersion (%)
		Pt	Au	
$Au_{3.1}$@1ML-Pt	3	0.4	0.7	-
$Au_{4.2}$@1ML-Pt	3	0.5	1	-
$Au_{5.3}$@1ML-Pt	3	0.9	2.8	91.9
$Au_{6.5}$@1ML-Pt	3	0.9	3.0	-
$Au_{5.3}$@0.5ML-Pt	1	0.4	2.8	100
$Au_{5.3}$@2ML-Pt	8	1.9	2.8	54.8
2.7 nm-Pt	-	3.5	-	42.1

Supplementary References,

[1] Kleis, J. et al. Finite size effects in chemical bonding: from small clusters to solids. *Catal. Lett.* 141, 1067–1071, (2011).

[2] Wang, H. et al. Precisely-controlled synthesis of Au@Pd core–shell bimetallic catalyst via atomic layer deposition for selective oxidation of benzyl alcohol. *J. Catal.* 324, 59-68, (2015).

[3] Enache, D. et al. Solvent-free oxidation of primary alcohols to aldehydes using Au-Pd/TiO_2 catalysts. *Science* 311, 362-365, (2006).

[4] Silva, T. A. G. et al. Volcano-like Behavior of Au-Pd Core-shell Nanoparticles in the Selective Oxidation of Alcohols. *Sci. Rep.* 4, 5766, (2014).

[5] Chen, Y. et al. Solvent-free aerobic oxidation of benzyl alcohol over Pd monometallic and Au–Pd bimetallic catalysts supported on SBA-16 mesoporous molecular sieves. *Appl. Catal. A* 380, 55-65, (2010).

[6] Chen, Y. et al. Formation of monometallic Au and Pd and bimetallic Au–Pd nanoparticles confined in mesopores via Ar glow-discharge plasma reduction and their catalytic applications in aerobic oxidation of benzyl alcohol. *J. Catal.* 289, 105-117, (2012).

[7] Zhu, X. et al. Optimising surface *d* charge of AuPd nanoalloy catalysts for enhanced catalytic activity. *Nat. Commun.* 10, 1428, (2019).

[8] Ines et al. Selective oxidation of benzyl alcohol using in situ generated H_2O_2 over hierarchical Au-Pd titanium silicalite catalysts. *Catal. Sci. Technol.* 3, 2425-2434, (2013).

[9] Hou, W. et al. Alcohol oxidations in aqueous solutions using Au, Pd, and bimetallic AuPd nanoparticle catalysts. *J. Catal.* 253, 22-27, (2008).

[10] Hutchings et al. Direct synthesis of hydrogen peroxide and benzyl alcohol oxidation using Au-Pd catalysts prepared by sol immobilization. *Langmuir* 26, 16568-16577, (2010).

[11] Yu, S. et al. Au–Pd nanoparticles on layered double hydroxide: Highly active catalyst for aerobic oxidation of alcohols in aqueous phase. *Catal. Commun.* 18, 142-146, (2012).

[12] Dan, I. E. et al. Solvent-free oxidation of benzyl alcohol using titania-supported gold–palladium catalysts: Effect of Au–Pd ratio on catalytic performance. *Catal. Today* 122, 407-411, (2007).

[13] Dimitratos, N. et al. Pd and Pt catalysts modified by alloying with Au in the selective oxidation of alcohols. *J. Catal.* 244, 113-121, (2006).
